# TOMBU and COMBU as Novel Uronium-Type Peptide Coupling Reagents Derived from Oxyma-B

**DOI:** 10.3390/molecules191118953

**Published:** 2014-11-18

**Authors:** Yahya E. Jad, Sherine N. Khattab, Beatriz G. de la Torre, Thavendran Govender, Hendrik G. Kruger, Ayman El-Faham, Fernando Albericio

**Affiliations:** 1 Catalysis and Peptide Research Unit, School of Health Sciences, University of Kwazulu-Natal, Durban 4001, South Africa; E-Mails: yahyajad@yahoo.com (Y.E.J.); bgarcia@yachaytech.edu.ec (B.G.T.); govenderthav@ukzn.ac.za (T.G.); kruger@ukzn.ac.za (H.G.K.); 2 Department of Chemistry, Faculty of Science, Alexandria University, P.O. Box 426, Ibrahimia, Alexandria 21321, Egypt; E-Mail: sh.n.khattab@gmail.com; 3 School of Chemistry, Yachay Tech, Yachay City of Knowledge, 100119-Urcuqui, Ecuador; 4 Department of Chemistry, College of Science, King Saud University, P.O. Box 2455, Riyadh 11451, Saudi Arabia; 5 School of Chemistry and Physics, University of KwaZulu-Natal, Durban 4001, South Africa; 6 Institute for Research in Biomedicine (IRB Barcelona), Baldiri Reixac 10, Barcelona, 08028, Spain; 7 CIBER-BBN, Networking Centre on Bioengineering, Biomaterials and Nanomedicine, Barcelona Science Park, Barcelona 08028, Spain; 8 Department of Organic Chemistry, University of Barcelona, Barcelona, 08028, Spain

**Keywords:** coupling reagents, solid-phase peptide synthesis, peptide synthesis, Oxyma-B, amino acids

## Abstract

Here we describe two novel uronium salts, TOMBU and COMBU, derived from the recently described Oxyma-B for use in peptide bond synthesis. These coupling reagents are more stable than COMU in DMF. Furthermore, using various peptide synthetic models in solution and solid-phase synthesis, we reveal that they show better performance than HBTU in terms of preserving chiral integrity and coupling yields, but slightly worse performance than COMU.

## 1. Introduction

**Figure 1:**
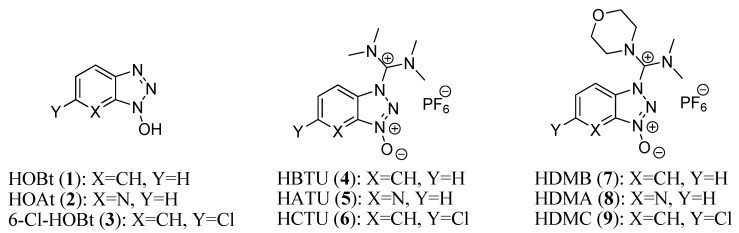
Structure of 1-hydroxybenzotriazoles and their aminium salts.

The formation of peptide bonds (also known as amide bonds) is dependent on the coupling reagent, which reacts with the carboxylic acid group to form an active species. This active species can be previously prepared, isolated, and purified, or prepared *in situ*. It then reacts with an amine to form the peptide bond [1,2,3,4,5,6,7]. Traditionally, carbodiimides were used as coupling reagents. The addition of phenols and *N*-hydroxy derivatives [8] to carbodiimides during the coupling reaction leads to the suppression of racemization and an enhancement of yield as a result of the inhibition ofside-reactions such as *N*-acylurea formation. 1-Hydroxybenzotriazole (HOBt, **1**) [9] was reported in the 1970s and has been used for decades in mostcoupling reactions in combination with carbodiimides. Later, the more reactive 7-aza-1-hydroxybenzotriazole (HOAt, **2**) [10] and 6-chloro-1-hydroxybenzotriazole (6-Cl-HOBt, **3**) [11] were added to the arsenal of *N*-hydroxy additives used with carbodiimides. However, the need for stronger activating reagents led to the replacement of carbodiimides with stand-alone coupling reagents (such as aminium/uronium and phosphonium ones). Aminium salts, such as HBTU (**4**), [12,13] HATU (**5**), [14,15] and HCTU (**6**), [16] are probably the most widely used and the most powerful examples. [17] Aminium salts **4**–**6** bear a tetramethyl moiety on their carbocation skeletons, and additives **1**–**3** are utilized as leaving groups for **4**–**6**, respectively. In 2007, our group reported that the replacement of one of the two dimethylamino groups by a morpholine group on the carbon skeleton [HDMB (**7**), HDMA (**8**) and HDMC (**9**)] increased the reactivity of these coupling reagents with respect to those containing the classical tetramethylimmonium or tetramethylaminium salts [18,19] (Figure 1).

**Figure 2:**
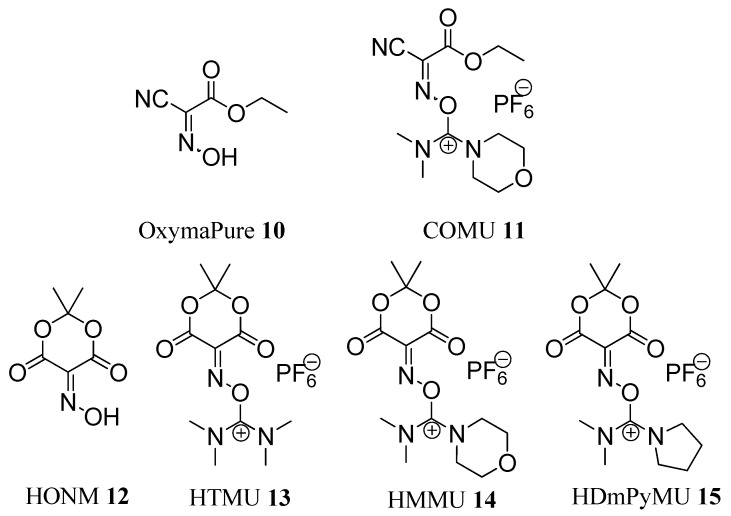
Structures of OxymaPure (**10**), COMU (**11**), HONM (**12**), and the uronium salts of HONM **13**–**15**.

Later, in 2009, OxymaPure (**10**) and its dimethylmorpholineuronium salt COMU (**11**) were reported as an efficient additive and coupling reagent, respectively, for peptide synthesis (Figure 2). OxymaPure and COMU displayed a remarkable efficiency to inhibit racemization and an impressive coupling efficiency, superior to the efficiencies shown by HOBt derivatives and comparable to those of HOAt derivatives. In addition, they had a lower risk of explosion than that of benzotriazole derivatives [20,21,22,23,24].

More recently, in 2010, a new family of uronium salts [HTMU (**13**), HMMU (**14**), and HDmPyMU (**15**)] based on isonitroso Meldrum’s acid (HONM, **12**) was reported as stand-alone coupling reagents [25] (Figure 2). HONM (**12**) shows structural similarities to OxymaPure (**10**), except that it has a special orientation of the carbonyl moiety and can therefore play an assisted basic catalytic role during the coupling reaction. While HONM (**12**)cannot be used as an additive for the carbodiimide, because it reacts with this functional group to form a nonreactive intermediate [25], its uronium salts **13**–**15** showed increased reactivity when compared with classical coupling reagents, especially during acylation of non-hindered poor nucleophiles, such as *p*-chloroaniline [25].

**Figure 3:**
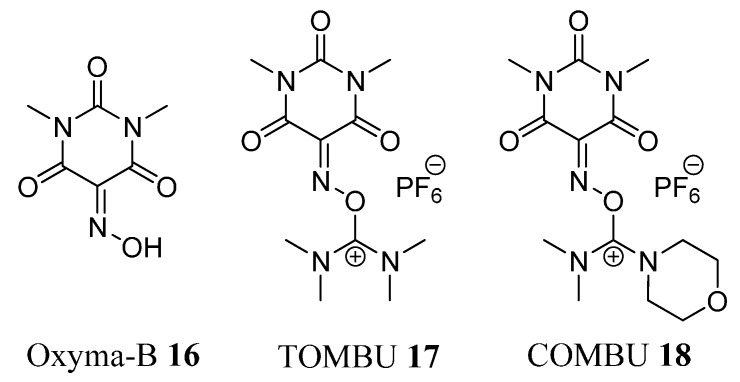
Structures of Oxyma-B (**16**) and its uronium salts.

More recently, we have reported 5-(hydroxyimino)-1,3-dimethylpyrimidine-2,4,6(1*H*,3*H*,5*H*)-trione (Oxyma-B, **16**) as an excellent additive for the suppression of racemization during peptide synthesis [26]. Oxyma-B, which has the same carbonyl moiety structure in which the oxime group is flanked between the two carbonyl groups as in HONM, is less reactive than HONM, thus allowing its use in combination with carbodiimides. In addition, Oxyma-B shows superior performance as a racemization suppressor than OxymaPure and HOAt in both stepwise and segment coupling in solid- and solution-phase peptide synthesis [26]. Here we introduce TOMBU (**17**) and COMBU (**18**), novel uronium-type coupling reagents involving Oxyma-B (**16**) as a leaving group (Figure 3).

## 2. Results and Discussion

### 2.1. Preparation of TOMBU and COMBU

**Scheme 1:**
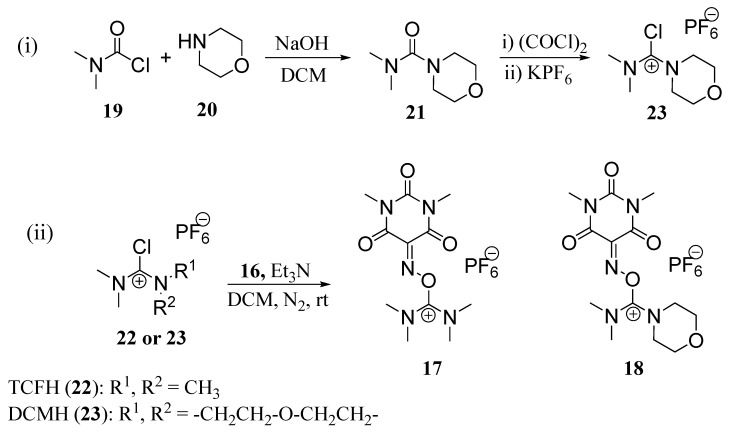
Synthetic scheme for preparing TOMBU (**17**) and COMBU (**18**).

Uronium salts **17** and **18**, both based on Oxyma-B, were prepared following a reported method [18,19,21,22,27].Thus, for instance the reaction of *N*,*N*-dimethylcarbamoyl chloride (**19**) with (**20**) renders dimethylmorpholineurea (**21**). This compound was then treated with oxalyl chloride to yield the corresponding chloride salt, which was stabilized by the formation of a PF_6_ salt (**23**). For the preparation of **17**, TCFH **22** was obtained from Iris Biotech GmbH and used without further purification. The chloride salts were subsequent reacted with Oxyma-B (**16**) under N_2_ atmosphere in DCM and in the presence of Et_3_N at room temperature to afford the desired compounds **17** and **18** as crystalline and stable solids (Scheme 1).

Carpino *et al*. [28] reported that HBTU (**4**) and HATU (**5**) exist in the N*-*form (guanidinium salt) instead of the O-form (uronium salt) and that the two forms can easily be distinguished by means of ^13^C-NMR spectroscopy. The carbocationic carbon of the *N-*form appears at 151–152 ppm while that of the O-form appears at 161–162 ppm [29,30]. In the case of TOMBU (**17**) and COMBU (**18**), their carbocationic carbons appeared at 162.1 and 162.5 ppm, respectively. This observation indicates that the new reagents were in O-form and therefore may be more reactive than the classical benzotriazole derivatives since this form is usually more reactive than the N-form [29].

### 2.2. Solubility

The solubility of the coupling reagents is crucial in determining the suitability of coupling reagents, especially in the automatic mode. Thus, the solubility of the novel coupling reagents was evaluated in DMF. COMBU (**18**) was more soluble than TOMBU (**17**) in DMF (entry 5 *vs.* 4, Table 1). This finding was expected since the presence of a morpholine ring on the carbon skeleton enhances the solubility of the compound. However, COMBU (**18**) showed lower less solubility in DMF than COMU. Again, this was also expected because of the presence of the ester group in the leaving group moiety.

**Table 1:** Molar solubility of uronium/aminium-type coupling reagents in DMF.

Entry	Coupling Reagent	Molarity
**1**	HBTU (**4**)	0.46
**2**	HATU (**5**)	0.45
**3**	COMU (**11**)	1.38
**4**	TOMBU (**17**)	0.28
**5**	COMBU (**18**)	0.71

### 2.3. Hydrolytic Stability

The stability of coupling reagents is a basic feature for determining their suitability in automatic mode peptide synthesis since stock solutions of these reagents in DMF are commonly used for several days. The dimethylmorpholinouronium salt COMBU (**18**) was less stable than the corresponding tetramethyl derivative TOMBU (**17**). Furthermore, TOMBU (**17**) and COMBU (**18**) showed greater stability than COMU (**11**) and less stability than HBTU (**4**) and HATU (**5**), as shown in Table 2. We can conclude from this study that aminium salts (also known as guanidinium salts), such as HBTU (**4**) and HATU (**5**), are considerably more stable than uronium ones.

**Table 2:** Closed vials hydrolytic stability of uronium/aminium-type coupling reagents in DMF ^[a]^.

Entry	Coupling Reagent	2 min	1 h	4 h	6 h	24 h	48 h
**1**	HBTU (**4**)	100	100	100	100	100	100
**2**	HATU (**5**)	100	100	100	100	100	99
**3**	COMU (**11**)	93	79	45	32	3	0
**4**	TOMBU (**17**)	97	95	89	84	35	13
**5**	COMBU (**18**)	88	85	70	61	10	0

^[a]^ 0.2 M solutions of each coupling reagent in DMF were used.

### 2.4. Racemization Control

There are two key parameters in the evaluation of a new coupling reagent, namely racemization and coupling performance. In order to examine the configuration retention induced by the new coupling reagents, the novel uronium coupling reagents were tested and compared with HBTU (**4**), HATU (**5**), and COMU (**11**) using the previously studied peptide models, namely the stepwise coupling of Z-Phg-Pro-NH_2_(**24**) and segment coupling of Z-Phe-Val-Pro-NH_2_(**25**) [20,21,25]. In the first model (**24**), the α-phenyl moiety in Phg ensured high sensitivity toward racemization because of the high stability of the counter anion. Oxyma-B-based uronium salts **17** and **18** showed better performance in reducing racemization than HBTU (**4**) and HATU(**5**) and similar performance to COMU (**11**). Moreover, TOMBU (**17**) gave a better conversion yield than HBTU (**4**) and HATU (**5**). However, COMBU (**18**) gave a slightly lower conversion yield than **17** (Table 3).

**Table 3:** Yield and racemization during the formation of Z-Phg-Pro-NH_2_ (**24**) (solution-phase synthesis) ^[a]^.

Entry	Coupling Reagent	Yield (%)^[b]^	DL (%)^[c]^
**1**	HBTU (**4**)	93	7
**2**	HATU (**5**)	96	4
**3**	COMU (**11**)	98	1
**4**	TOMBU (**17**)	99	1
**5**	COMBU (**18**)	93	1

^[a]^ Couplings were performed without pre-activation using two equiv. of DIEA in DMF at room temperature; ^[b]^ Conversion yield of the product (LL+ DL) was calculated by HPLC. The HPLC traces showed some of the starting material (unreacted acid, Z-Phg-OH) at *t*_R_ 8.8 min in ratio 1.6%–6.8%. Retention times of Z-Phg-OH and Z-Phg-Pro-NH_2_ were identified by injection of a pure sample; ^[c]^ Retention times for each epimer were identified after co-injection with pure LL and DL samples onto reverse-phase HPLC using a linear gradient of 25% to 50% 0.1% TFA in CH_3_CN/ 0.1% TFA in H_2_O over 15 min, detection at 220 nm and a Phenomex C_18_ (3 μm, 4.6 × 50 mm) column, *t*_R_(LL) = 6.4 min, *t*_R_ (DL) = 6.8 min.

The second model was the segment coupling (2+1) of dipeptide Z-Phe-Val-OH onto H-Pro-NH_2_ to afford Z-Phe-Val-Pro-NH_2_ (**25**). This model is known to give higher racemization levels than the previous stepwise coupling model because oxazolone formation during the activation of dipeptide is promoted as a result of the electron-donating effect of the *N*-aminoacyl substitution [6]. In the segment coupling model (**25**), the best results were obtained with HATU (**5**), which gave the highest yield and the lowest racemization. However, TOMBU (**17**) and COMBU (**18**) showed better performance in reducing the racemization than HBTU (**4**). Furthermore, TOMBU (**17**) gave lower racemization than COMU (**11**) (Table 4).

**Table 4:** Yield and racemization during the formation of Z-Phe-Val-Pro-NH_2_ (**25**) (solution-phase synthesis) ^[a]^.

Entry	Coupling Reagent	Yield (%) ^[b]^	LDL (%) ^[c]^
**1**	HBTU (**4**)	97	30
**2**	HATU (**5**)	98	7
**3**	COMU (**11**)	98	14
**4**	TOMBU (**17**)	92	13
**5**	COMBU (**18**)	91	17

^[a]^ Couplings were performed without pre-activation using two equiv. of DIEA in DMF at room temperature; ^[b]^ Conversion yield of the product (LLL+ LDL) was calculated from HPLC. The HPLC traces showed some of the starting material (unreacted acid, Z-Phe-Val-OH) at *t*_R_ 7.8 min.in ratio 1.5%–7.5%.Retention times of Z-Phe-Val-OH and Z-Phe-Val-Pro-NH_2_ were identified by injection of a pure sample; ^[c]^ Retention times for each epimer were identified after co-injection with pure LLL and LDL samples onto reverse-phase HPLC using a linear gradient of 30% to 60% 0.1% TFA in CH_3_CN/0.1% TFA in H_2_O over 15 min, detection at 220 nm and a Phenomex C_18_ (3 μm, 4.6 × 50 mm) column, *t*_R_(LLL) = 5.8 min, *t*_R_(LDL) = 6.9 min.

### 2.5. Coupling Efficiency

To demonstrate the efficiency of these novel coupling reagents in peptide synthesis, Aib-enkephaline pentapeptide (**26**) was used as a model for SPPS [19,21,22,25,31]. Non-incorporation of one Aib residue, giving des-Aib, is the most important side reaction and is caused by slow incorporation of the Aib residue because of its sterically hindered nature. Therefore, clear differences among the performances of coupling reagents can be obtained. Aib-enkephaline pentapeptide (**26**) was manually assembled stepwise on Fmoc-RinkAmide-AM-PS-resin by means of a 30 min coupling (except for Aib-Aib where a 30 min double coupling was applied) with an excess of 3 equiv. of Fmoc-amino acid/coupling reagent and 6 equiv. of DIEA. As shown in Table 5, TOMBU (**17**) and COMBU (**18**) showed better performance than HBTU (**4**) and poorer performance than HATU (**5**) and COMU (**11**) as shown in Table 5.

**Table 5:** Percentage purity of pentapeptide **26** (H-Tyr-Aib-Aib-Phe-Leu-NH_2_) during solid-phase assembling, in the presence of the side product forming tetrapeptide des-Aib (H-Tyr-Aib-Phe-Leu-NH_2_) ^[a]^.

Entry	Coupling Reagent	Base (equiv.)	Pentapeptide (%)	des-Aib (%) ^[b]^
**1**	HBTU (**4**)	DIEA (2)	53	47
**2**	HATU (**5**)	DIEA (2)	98	2
**3**	COMU (**11**)	DIEA (2)	99	1
**4**	TOMBU (**17**)	DIEA (2)	90	10
**5**	COMBU (**18**)	DIEA (2)	82	18
**6**	COMBU (**18**)	DIEA (2) ^[c]^	84	16

^[a]^ 1–2 min pre-activation and 30 min coupling times were generally applied, except for Aib-Aib (30 min double coupling); ^[b]^ Deletion tetrapeptide (des-Aib) was identified by peak overlap in HPLC with an authentic sample obtained in solid phase. The crude H-Tyr-Aib-Aib-Phe-Leu-NH_2_ was analyzed by reverse-phase HPLC using linear gradient of 30% to 60% 0.1% TFA in CH_3_CN/0.1% TFA in H_2_O over 15 min, detection at 220 nm and a Phenomex C_18_ (3 μm, 4.6 × 50 mm) column, *t*_R_ = 6.68 (pentapeptide), 6.78 (des-Aib) min; ^[c]^ Fmoc-amino acids were pre-activated with only 1 equiv. of DIEA for 15–30 s, with addition of another 1 equiv. onto the resin after the first addition.

## 3. Experimental Section

### 3.1. Materials

The solvents used were of HPLC reagent grade. Chemicals and amino acid derivatives were purchased from Sigma-Aldrich (Steinheim, Germany), Fluka (Steinheim, Germany), Gl Biochem (Shanghai) Ltd. (Shanghai, China), Iris Biotech GmbH (Marktredwitz, Germany), or Merck Millipore (Bedford, MA, USA). The following coupling reagents were used HBTU (Luxembourg Biotech. Ltd, Batch number 1103193083), HATU (Luxembourg Biotech. Ltd, Batch number 50918017) and COMU (Luxembourg Biotech. Ltd., Batch number 1302108000). Melting points were determined with a Buchi B-540 apparatus (BUCHI Labortechnick GmbH, Essen, Germany) and are uncorrected. NMR spectra (^1^H-NMR and ^13^C-NMR) were recorded on a Burker AVANCE III 400 MHz spectrometer (Rheinstetten, Germany). Chemical shift values are expressed in ppm downfield from TMS as an internal standard. Follow-up of the reactions and initial confirmation of the purity of the compounds was performed by TLC on silica gel-protected aluminum sheets (Type 60 GF254, Merck Millipore), and the spots were detected by exposure to UV-lamp at λ 254 nm for a few seconds. Analytical HPLC was performed on an Agilent 1100 system (Kyoto, Japan), and Chemstation software was used for data processing. LC-MS was performed on a Shimadzu 2020 UFLC-MS instrument (Kyoto, Japan) using an YMC-Triart C_18_ (5 μm, 4.6 × 150 mm) column, and data processing was carried out by LabSolution software. Buffer A: 0.1% formic acid in H_2_O; and buffer B: 0.1% formic acid in CH_3_CN. High-resolution mass spectrometric data were obtained using a Brukermicr OTOF-Q II instrument (Bermen, Germany) operating at room temperatures and a sample concentration of approximately 1 ppm. 

### 3.2. Synthesis of N,N-Dimethylmorpholine-4-carboxamide (DMU)

Morpholine (0.5 mol) was dissolved in DCM (300 mL) and 10% NaOH (300 mL). Dimethyl carbamoyl chloride (0.6 mol) in 200 mL of DCM was then added over 10 min. When the addition was completed, the mixture was stirred for 3 h at r.t. The organic layer was collected, and the aqueous layer was washed with DCM (100 mL). The combined DCM solution was washed with a saturated solution of NaCl (2 × 100 mL). Finally, the organic solvent was dried over anhydrous MgSO4 and filtered. The solvent was then removed under reduced pressure to give an oily residue. The product was distilled and collected at bp 127–129 °C as colorless oil in 93% yield. ^1^H-NMR (CDCl_3_): δ2.72 (s, 6H, 2 CH_3_), 3.08–3.11 (m, 4H, 2CH_2_), 3.54–3.57 (m, 4H, 2CH_2_) ppm. ^13^C-NMR (CDCl_3_): δ38.0, 47.1, 66.3, 164.3 ppm.

### 3.3. Synthesis of 4-[(Dimethyamino)chloromethylene]morpholin-4-iminium Hexafluorophosphate (DCMH)

Oxalyl chloride (100 mmol) in dry DCM (100 mL) was added dropwise to a solution of urea derivative (100 mmol) in dry DCM (200 mL) at r.t. over 5 min. The reaction mixture was stirred under reflux conditions for 3 h, and the solvent was removed under vacuum. The residue was washed with anhydrous ether (2 × 100 mL) and then bubbled with N_2_ to remove the excess of ether. The residue was highly hygroscopic and was therefore dissolved directly in DCM, and a saturated aqueous potassium hexafluorophosphate (100 mmol in 50 mL H_2_O) solution was then added at r.t. with vigorous stirring for 10–15 min. The organic layer was collected, washed once with water (100 mL), dried over anhydrous MgSO_4_, and filtered. The solvent was removed under reduced pressure to give a white solid which recrystallized from acetonitrile-diethyl ether to give white crystals in 89% yield; m.p. 94–95°C; ^1^H-NMR (CD_3_CN): δ 3.39 (s, 6H; 2CH_3_), 3.75 (m, 4H; 2CH_2_), 3.86 ppm (m, 4H; 2CH_2_); ^13^C-NMR (CD_3_CN): δ 44.5, 52.8, 66.0, 162.9 ppm.

### 3.4. General Procedure for the Preparation of Uronium-Type Coupling Reagents Based on Oxyma-B

The chloroformamidinium salt (20 mmol) was added to a stirring solution of Oxyma-B (20 mmol) and triethylamine (20 mmol) in dry DCM (50 mL) at 0 °C. The reaction mixture was stirred at r.t. overnight, and then filtered and washed with DCM (10 mL). The residue was recrystallized from acetonitrile-diethyl ether.

#### 3.4.1. *N*-((1,3-Dimethyl-2,4,6-trioxotetrahydropyrimidin-5(6*H*)-ylideneaminooxy)(dimethylamino) methylene)-*N*-methylmethanaminiumhexafluorophosphate (TOMBU, **17**)

The product was obtained as a white solid in 90% yield (7.8 g); m.p. 199–200 °C with decomposition; ^1^H-NMR (CD_3_CN)δ 3.07 (s, 12H, 4 CH_3_), 3.2 (s, 3H, CH_3_), 3.24 (s, 3H, CH_3_). ^13^C-NMR (CD_3_CN): δ 27.9, 28.3, 40.2, 141.0, 149.5, 151.9, 155.4, 162.1.HRMS (ESI+) *m/z* calcd forC_11_H_18_N_5_O_4_^+^: [M]^+^ 284.1353; found [M]^+^ 284.1356.

#### 3.4.2. 4-((1,3-Dimethyl-2,4,6-trioxotetrahydropyrimidin-5(6*H*) ylideneaminooxy)(dimethylamino) methylene)morpholin-4-ium hexafluorophosphate (COMBU, **18**)

The product was obtained as a pale yellow solid in 84% yield (8 g); m.p. 166–167 °C with decomposition, ^1^H-NMR (CD_3_CN)δ 3.14 (s, 6H, 2 CH_3_), 3.26 (s, 3H, CH_3_), 3.30 (s, 3H, CH_3_), 3.51–3.53 (m, 4H, 2CH_2_), 3.78–3.81 (m, 4H, 2CH_2_). ^13^C-NMR (CD_3_CN):δ 29.2, 29.6, 41.4, 50.5, 66.7, 142.1, 150.7, 153.2, 156.6, 162.5.HRMS (ESI+) *m/z* calcd for C_13_H_20_N_5_O_5_^+^: [M]^+ ^326.1459; found [M]^+^ 326.1469.

### 3.5. Solubility Test

One mL of DMF was taken, and known amounts of coupling agents were added with stirring until no more solid was soluble. In some cases, sonication was needed.

### 3.6. Hydrolytic Stability Test

One mL of 0.2 M solution of coupling reagent was stored in a closed HPLC vial. At each interval time, an aliquot (10 μL) of the solution was taken and diluted to 1 mL with a mixture of CH_3_CN, and 1 μL was injected immediately into a reverse-phase HPLC apparatus. Yields of coupling reagent were calculated according to the integration of the peak area at 220 nm of the signal associated with the coupling reagent with respect to the corresponding leaving group.

### 3.7. General Method for the Racemization Experiments

An acid (Z-Phg-OH or Z-Phe-Val-OH, 0.125 mmol of), H-Pro-NH_2_ (0.125 mmol) and DIEA (0.25 mmol) were dissolved in DMF (2 mL), and the solution was cooled in an ice bath and then treated with the corresponding coupling reagent (0.125 mmol). The mixture was stirred at 0 °C for 1 h and at r.t. overnight. An aliquot (10 μL) of the solution was then taken and diluted to 1 mL with a mixture of CH_3_CN/H_2_O (1:2), and 5 μL was injected into a reverse-phase HPLC apparatus.

#### 3.7.1. Z-Phg-Pro-NH_2_

A linear gradient of 25%–50% CH_3_CN/H_2_O and 0.1% TFA over 15 min was applied, with a flow rate of 1.0 mL/min and detection at 220 nm using a Phenomex C_18_ (3 μm, 4.6 × 50 mm) column, *t*_R_ (LL) = 6.4 min, *t*_R_ (DL) = 6.8 min, *t*_R_(Z-Phg-OH) = 8.8 min.

#### 3.7.2. Z-Phe-Val-Pro-NH_2_

A linear gradient of 30%–60% CH_3_CN/H_2_O and 0.1% TFA over 15 min was applied, with a flow rate of 1.0 mL/min and detection at 220 nm using a Phenomex C_18_ (3 μm, 4.6 × 50 mm) column, *t*_R_ (LLL) = 5.8 min, *t*_R_ (LDL) = 6.9 min, *t*_R_(Z-Phe-Val-OH) = 7.7 min.

### 3.8. Solid-Phase Synthesis of H-Tyr-Aib-Aib-Phe-Leu-NH_2_

The synthesis was carried out in a plastic syringe attached to a vacuum manifold so as to effect rapid removal of reagents and solvent. The Fmoc-RinkAmide-AM-PS resin (0.6 mmol g^−1^, 100 mg) was washed with DMF, DCM, and DMF (2 × 10 mL each) and then treated with 20% piperidine in DMF (10 mL) for 10 min. The resin was then washed with DMF, DCM, and DMF (2 × 10 mL each). The resin was then acylated with a solution of Fmoc-Leu-OH (3 equiv.), the corresponding coupling reagent (3 equiv.), and DIEA (6 equiv.) in DMF (0.5 mL, previously pre-activated). After peptide coupling, the resin was washed with DMF and then deblocked by treatment with 20% piperidine in DMF for 7 min. The resin was washed with DMF, DCM, and DMF (2 × 10 mL each), and then coupling with the next amino acid, as explained before, and deblocking were repeated to obtain the pentapeptide. The peptide was cleaved from the resin with TFA/H_2_O (9:1) at r.t. for 2 h. TFA was removed under nitrogen, and the crude peptide was purified with cold Et_2_O (3 × 10 mL) and lyophilized. The ratio of the penta- and tetra-peptide was determined by HPLC analysis using a Phenomex C_18_ (3 μm, 4.6 × 50 mm) column, with a linear gradient of 20% to 40% of 0.1% TFA in CH_3_CN/0.1% TFA in H_2_O over 15 min, flow rate = 1.0 mL·min^−1^, and detection at 220 nm. The *t*_R_ values for pentapepide and des-Aib were 6.68 min and 6.78 min, respectively. LC-MS showed the expected mass for the penta at *m*/*z* = 611.0 and also for des-Aib at *m*/*z* = 526.

## 4. Conclusions

In conclusion, we have described a new class of O-form uronium-type coupling reagents for peptide bond formation derived from Oxyma-B. Importantly, the novel coupling reagents showed higher stability than COMU in DMF, which is itsmain drawback, and better performance in terms of reducing racemization, better yields than HBTU, and slightly poorer yields than COMU. It is envisaged that these new members of the arsenal of coupling reagents will find applications in the construction of peptide bonds, mainly in an automatic mode to overcome the instability of COMU in DMF.

## Supplementary Materials

Supplementary materials can be accessed at: http://www.mdpi.com/1420-3049/19/11/18953/s1.

## Supplementary Files

Supplementary File 1

## References

[1] Albericio F., Carpino L.A., Gregg B.F. (1997). Coupling reagents and activation. Methods Enzymol.

[2] Humphrey J.M., Chamberlin A.R. (1997). Chemical synthesis of natural product peptides: Coupling methods for the incorporation of noncoded amino acids into peptides. Chem. Rev..

[3] Han S.-Y., Kim Y.-A. (2004). Recent development of peptide coupling reagents in organic synthesis. Tetrahedron.

[4] Montalbetti C.A.G.N., Falque V. (2005). Amide bond formation and peptide coupling. Tetrahedron.

[5] Valeur E., Bradley M. (2009). Amide bond formation: Beyond the myth of coupling reagents. Chem. Soc. Rev..

[6] El-Faham A., Albericio F. (2011). Peptide coupling reagents, more than a letter soup. Chem. Rev..

[7] Albericio F., Chinchilla R., Dodsworth D.J., Najera C. (2001). New trends in peptide couplingreagents. Org. Prep. Proced. Int..

[8] Subirós-Funosas R., Albericio F., El-Faham A. (2009). N-hydroxylamines for peptide synthesis. Patai’s Chemistry of Functional Groups.

[9] König W., Geiger R. (1970). Eine neue methode zur synthese von peptiden: Aktivierung der carboxylgruppe mit dicyclohexylcarbodiimid unter zusatz von 1-hydroxy-benzotriazolen. Chem. Ber..

[10] Carpino L.A. (1993). 1-Hydroxy-7-azabenzotriazole. An efficient peptide coupling additive. J. Am. Chem. Soc..

[11] Sabatino G., Mulinacci B., Alcaro M.C., Chelli M., Rovero P., Papini A.M. *Peptides 2002,* Proceedings of the Twenty-Seventh European Peptide Symposium.

[12] Knorr R., Trzeciak A., Bannwarth W., Gillessen D. (1989). New coupling reagents in peptide chemistry. Tetrahedron Lett..

[13] Dourtoglou V., Ziegler J.-C., Gross B. (1978). L’hexafluorophosphate de o-benzotriazolyl-n,n-tetramethyluronium: Un reactif de couplage peptidique nouveau et efficace. Tetrahedron Lett..

[14] Carpino L.A., El-Faham A., Albericio F. (1994). Racemization studies during solid-phase peptide synthesis using azabenzotriazole-based coupling reagents. Tetrahedron Lett..

[15] Carpino L.A., El-Faham A., Minor C.A., Albericio F. (1994). Advantageous applications of azabenzotriazole (triazolopyridine)-based coupling reagents to solid-phase peptide synthesis. J. Chem. Soc. Chem. Commun..

[16] Marder O., Shvo Y., Albericio F. (2002). HCTU and TCTU. New coupling reagents: Development and industrial aspects. Chim. Oggi-Chem. Today.

[17] Albericio F., Bofill J.M., El-Faham A., Kates S.A. (1998). Use of onium salt-based coupling reagents in peptide synthesis. J. Org. Chem..

[18] El-Faham A., Albericio F. (2007). Novel proton acceptor immonium-type coupling reagents: Application in solution and solid-phase peptide synthesis. Org. Lett..

[19] El-Faham A., Albericio F. (2008). Morpholine-based immonium and halogenoamidinium salts as coupling reagents in peptide synthesis. J. Org. Chem..

[20] Subirós-Funosas R., Prohens R., Barbas R., El-Faham A., Albericio F. (2009). Oxyma: An efficient additive for peptide synthesis to replace the benzotriazole-based HOBt and HOAt with a lower risk of explosion. Chemistry.

[21] El-Faham A., Funosas R.S., Prohens R., Albericio F. (2009). COMU: A safer and more effective replacement for benzotriazole-based uronium coupling reagents. Chemistry.

[22] El-Faham A., Albericio F. (2010). COMU: A third generation of uronium-type coupling reagents. J. Pept. Sci..

[23] Subiros-Funosas R., Khattab S.N., Nieto-Rodriguez L., El-Faham A., Albericio F. (2013). Advances in acylation methodologies enabled by Oxyma-based reagents. Aldrichim. Acta.

[24] Wehrstedt K.D., Wandrey P.A., Heitkamp D. (2005). Explosive properties of 1-hydroxybenzotriazoles. J. Hazard. Mater..

[25] El-Faham A., Subirós-Funosas R., Albericio F. (2010). A novel family of onium salts based upon isonitroso meldrum's acid proves useful as peptide coupling reagents. Eur. J. Org. Chem..

[26] Jad Y.E., Khattab S.N., de la Torre B.G., Govender T., Kruger H.G., El-Faham A., Albericio F. (2014). Oxyma-B, an excellent racemization suppressor for peptide synthesis. Org. Biomol. Chem..

[27] El-Faham A., Khattab S.N., Abdul-Ghani M., Albericio F. (2006). Design and synthesis of new immonium-type coupling reagents. Eur. J. Org. Chem..

[28] Abdelmoty I., Albericio F., Carpino L., Foxman B., Kates S. (1994). Structural studies of reagents for peptide bond formation: Crystal and molecular structures of HBTU and HATU. Lett. Pept. Sci..

[29] Carpino L.A., Imazumi H., El-Faham A., Ferrer F.J., Zhang C., Lee Y., Foxman B.M., Henklein P., Hanay C., Mügge C. (2002). The uronium/guanidinium peptide coupling reagents: Finally the true uronium salts. Angew. Chem. Int. Ed..

[30] Carpino L.A., Henklein P., Foxman B.M., Abdelmoty I., Costisella B., Wray V., Domke T., El-Faham A., Mugge C. (2001). The solid state and solution structure of HAPyU. J. Org. Chem..

[31] El-Faham A., Khattab S.N., Subiros-Funosas R., Albericio F. (2014). BOP-OXy, BOP-OBt, and BOP-OAt: Novel organophosphinic coupling reagents useful for solution and solid-phase peptide synthesis. J. Pept. Sci..

