# Supplementary material for: TOMBU and COMBU as Novel Uronium-Type Peptide Coupling Reagents Derived from Oxyma-B
Source: Molecules. 2014 Nov 18;19(11):18953–65. doi: 10.3390/molecules191118953 (PMC6271478; doi:10.3390/molecules191118953)
Supplement: Supplementary File 1 [file molecules-19-18953-s001.pdf]

Supplementary

Figure S1. ^{1}H-NMR of TOMBU.

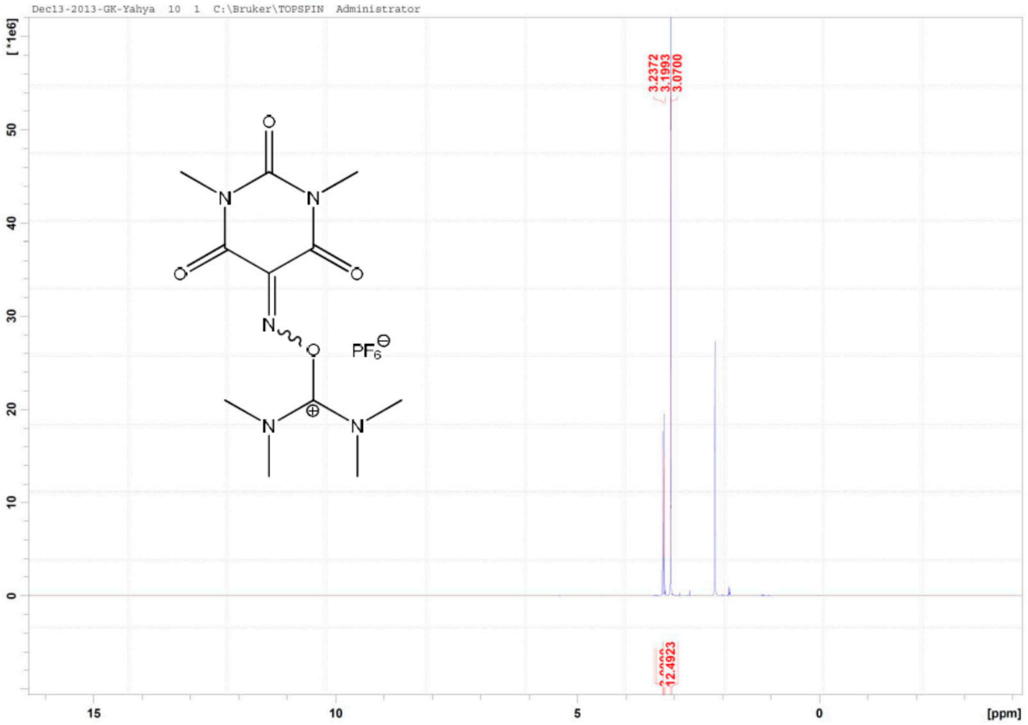

Figure S2. ^{1}H-NMR of TOMBU (Expansion for S1).

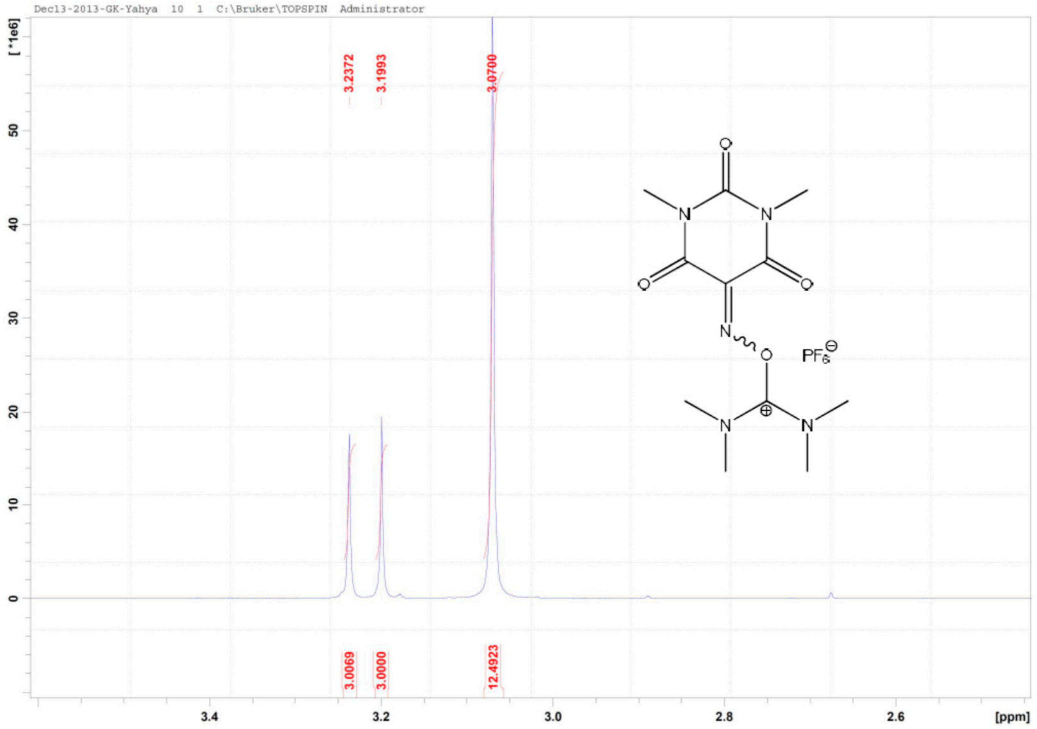

Figure S3. ^{13}C-NMR of TOMBU.

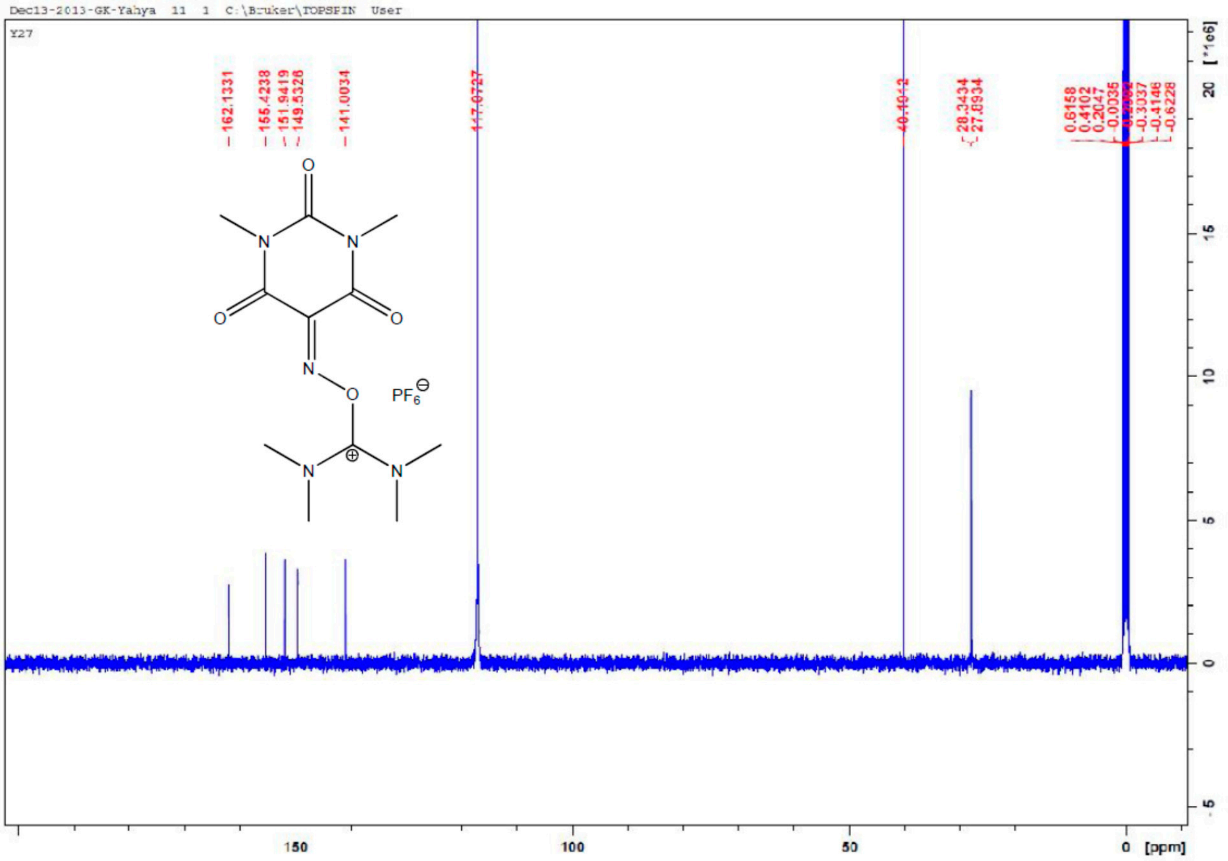

Figure S4. HRMS of TOMBU.

Display Report

Analysis Info

Analysis Name	D:\Data\YAHYA\10 October Y27 2nd trial_11_01_3519.d	Acquisition Date	10/10/2014 12:44:32 PM
Method	fia.m	Operator	BDAL@DE
Sample Name	10 October Y27 2nd trial	Instrument	micrOTOF-Q 10139
Comment			

Acquisition Parameter

Source Type	ESI	Ion Polarity	Positive	Set Nebulizer	4.0 Bar
Focus	Active	Set Capillary	4500 V	Set Dry Heater	220 °C
Scan Begin	150 m/z	Set End Plate Offset	-500 V	Set Dry Gas	8.0 l/min
Scan End	1600 m/z	Set Collision Cell RF	250.0 Vpp	Set Divert Valve	Source

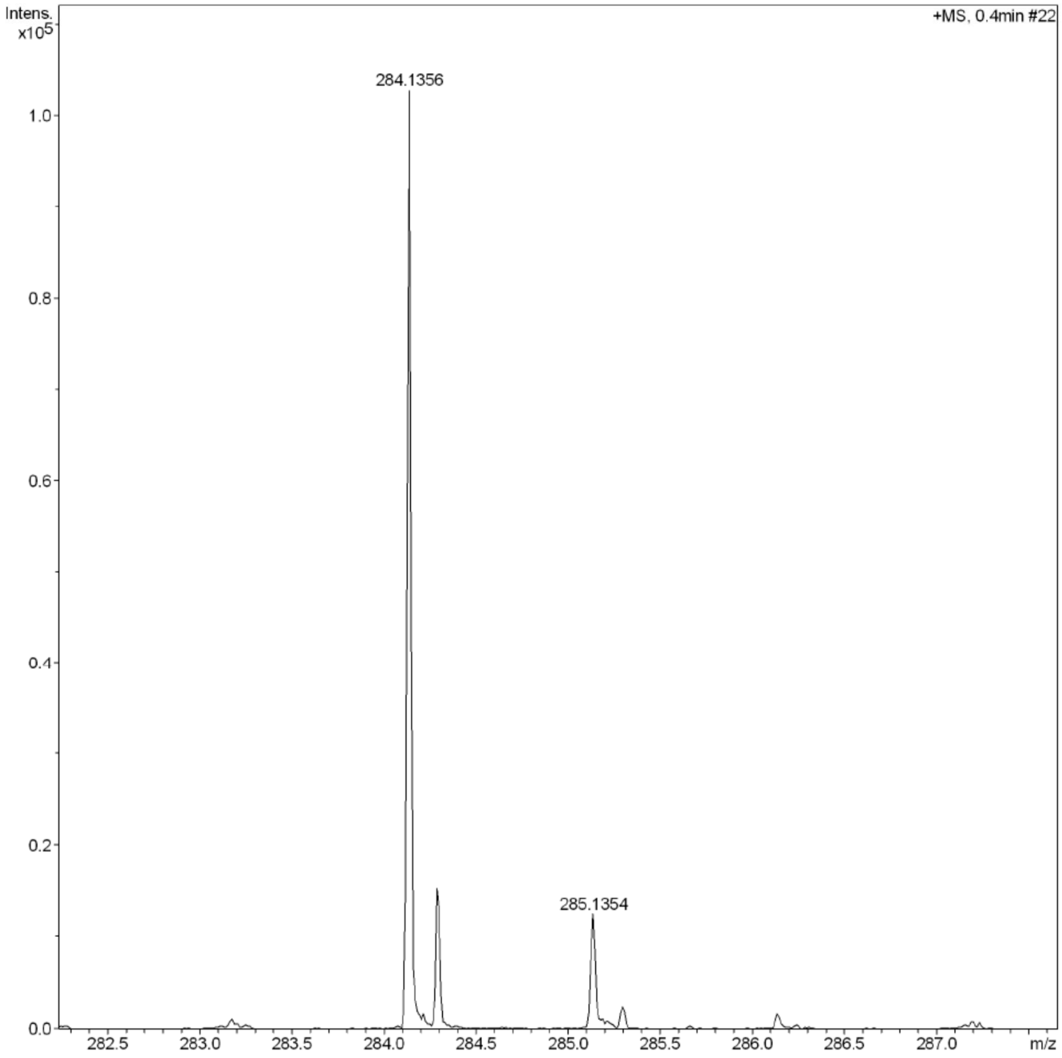

Figure S5. ^{1}H-NMR of COMBU.

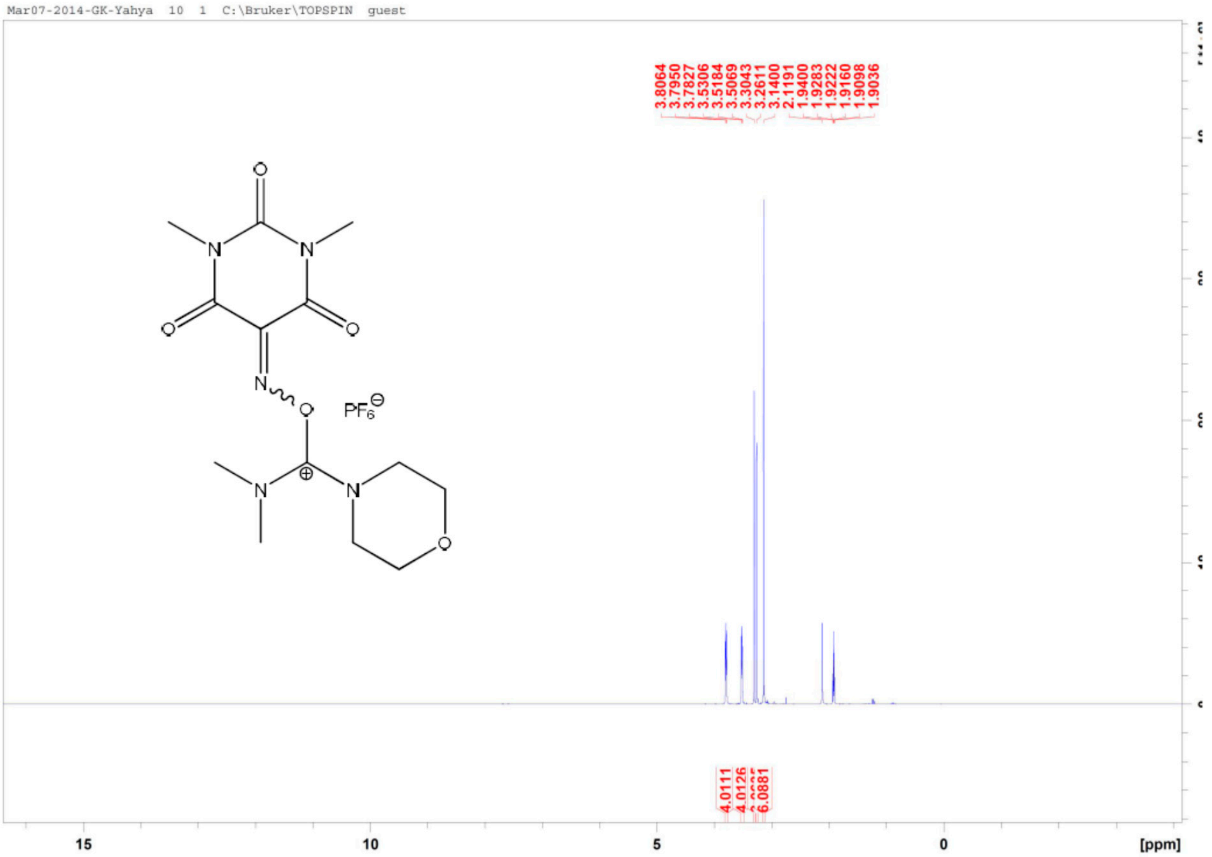

Figure S6. ^{1}H-NMR of COMBU (Expansion of S5).

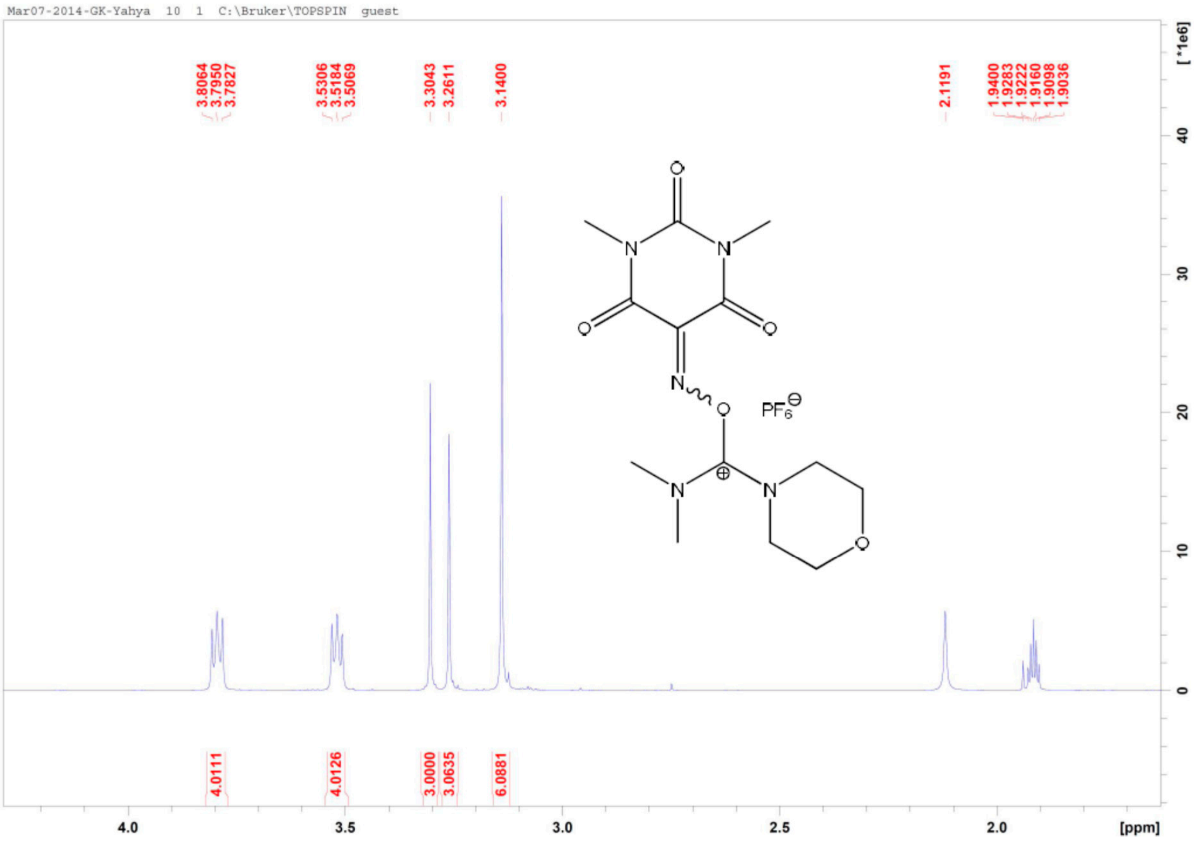

Figure S7. ^{13}C-NMR of COMBU.

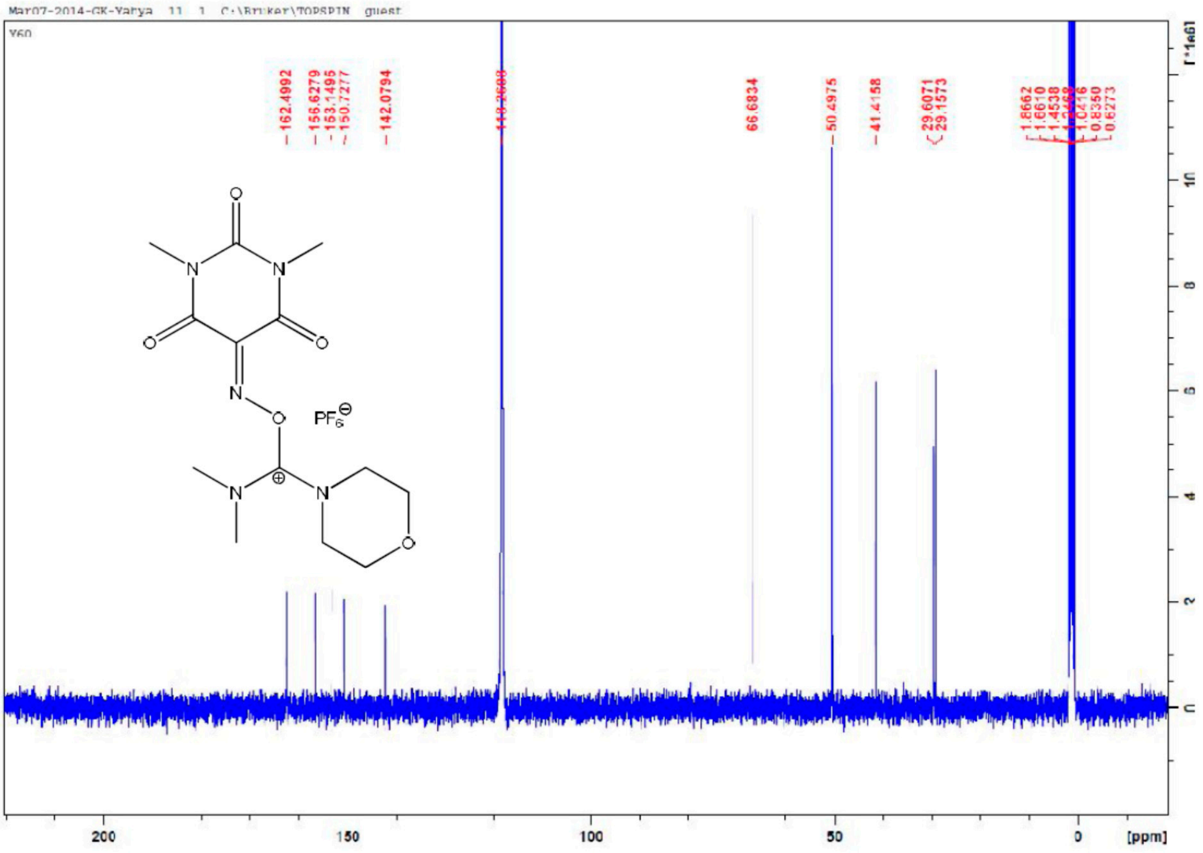

Figure S8. HRMS of COMBU.

Display Report

Analysis Info

Analysis Name	D:\Data\YAHYA\3 October Y60_3_01_3348.d	Acquisition Date	10/3/2014 1:13:35 PM
Method	fia.m	Operator	BDAL@DE
Sample Name	3 October Y60	Instrument	micrOTOF-Q 10139
Comment			

Acquisition Parameter

Source Type	ESI	Ion Polarity	Positive	Set Nebulizer	0.4 Bar
Focus	Not active	Set Capillary	4500 V	Set Dry Heater	200 °C
Scan Begin	250 m/z	Set End Plate Offset	-500 V	Set Dry Gas	4.0 l/min
Scan End	2300 m/z	Set Collision Cell RF	600.0 Vpp	Set Divert Valve	Source

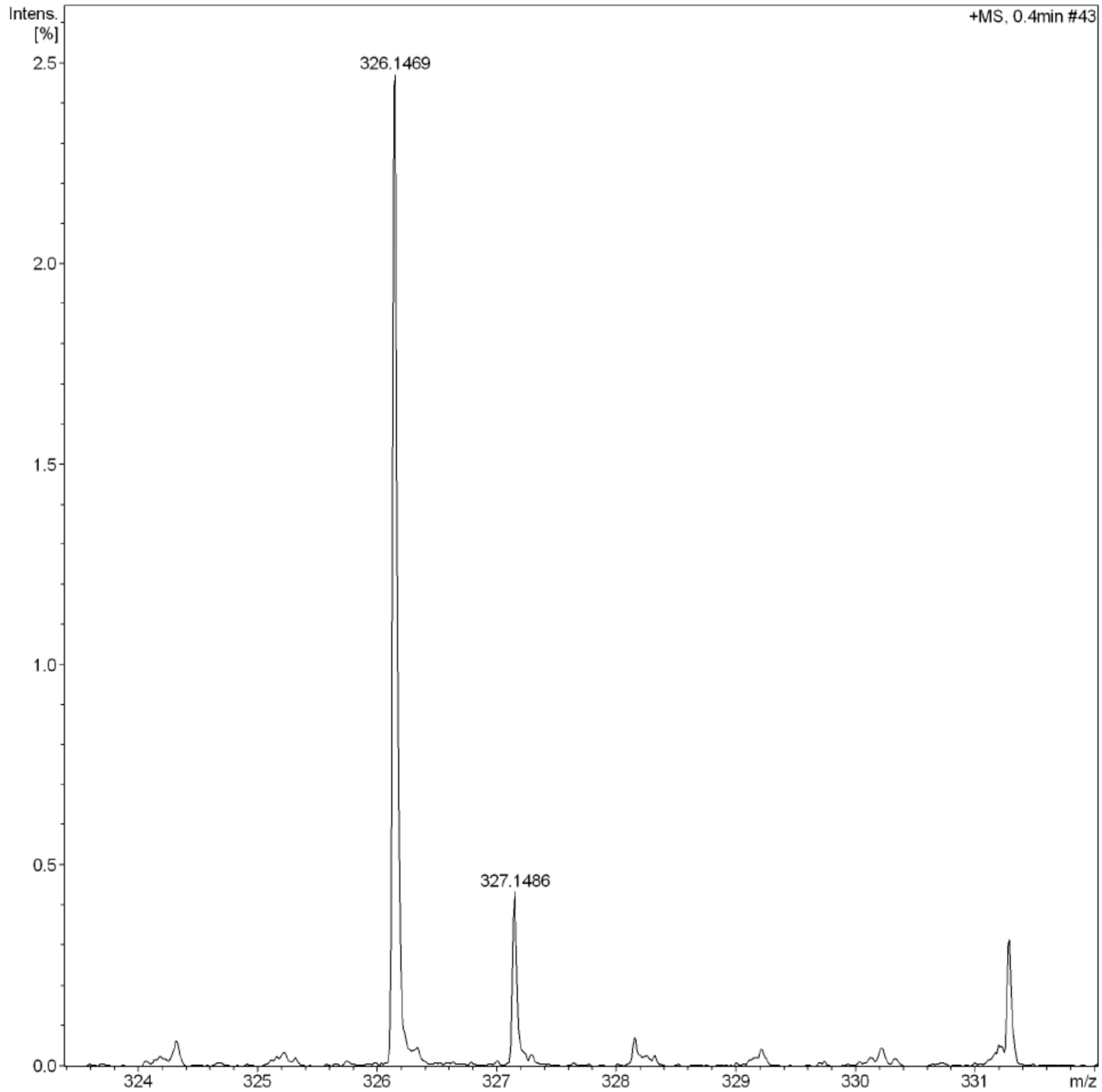

S9-S24 HPLC Analyses

1-Z-Phg-Pro-NH_2 model.

Figure S9. HBTU.

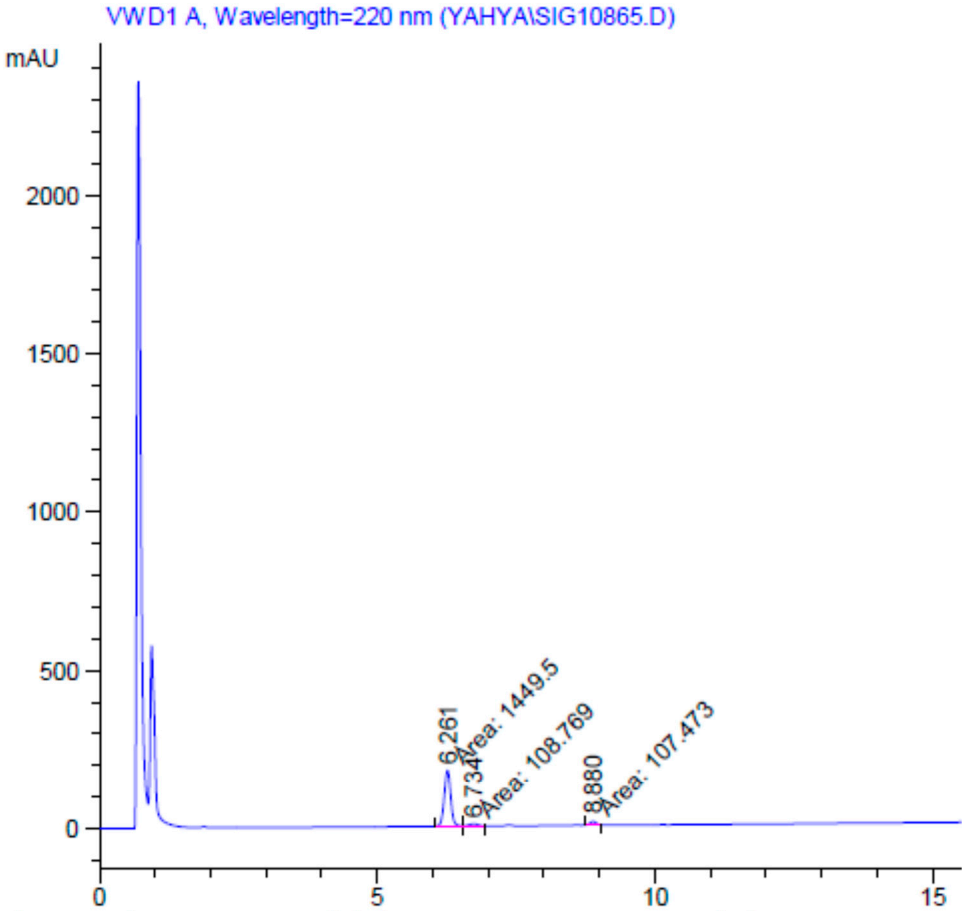

Peak #	RetTime [min]	Type	Width [min]	Area mAU *s	Height [mAU]	Area %
1	6.261	MF	0.1355	1449.49866	178.30231	87.0183
2	6.734	FM	0.2097	108.76921	8.64380	6.5298
3	8.880	MM	0.1483	107.47305	12.08140	6.4520
Totals :				1665.74092	199.02751	

Figure S10. HATU.

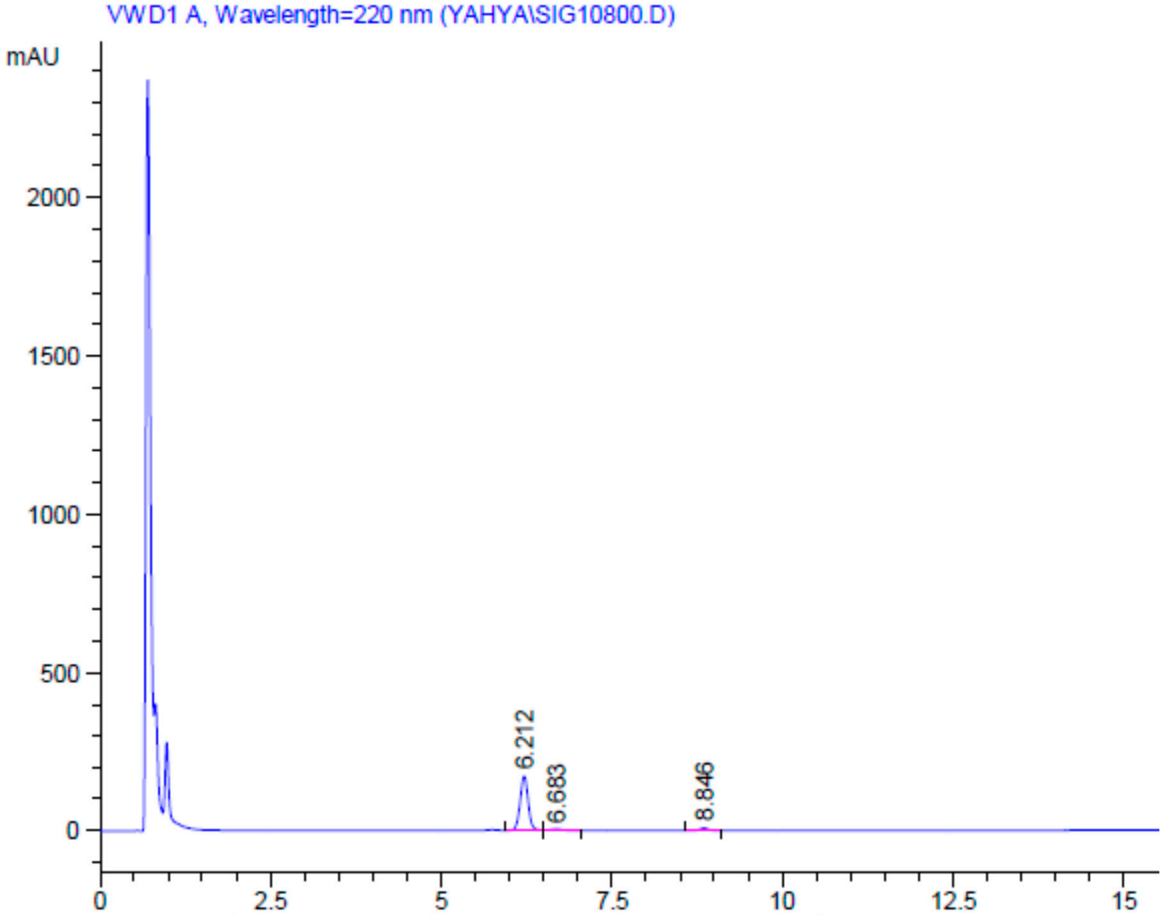

Peak #	RetTime [min]	Type	Width [min]	Area mAU *s	Height [mAU]	Area %
1	6.212	VV	0.1280	1408.26099	171.17581	92.0794
2	6.683	VB	0.1909	54.76175	4.34074	3.5806
3	8.846	VB	0.1330	66.37612	7.66997	4.3400

Totals : 1529.39886 183.18653

Figure S11. COMU.

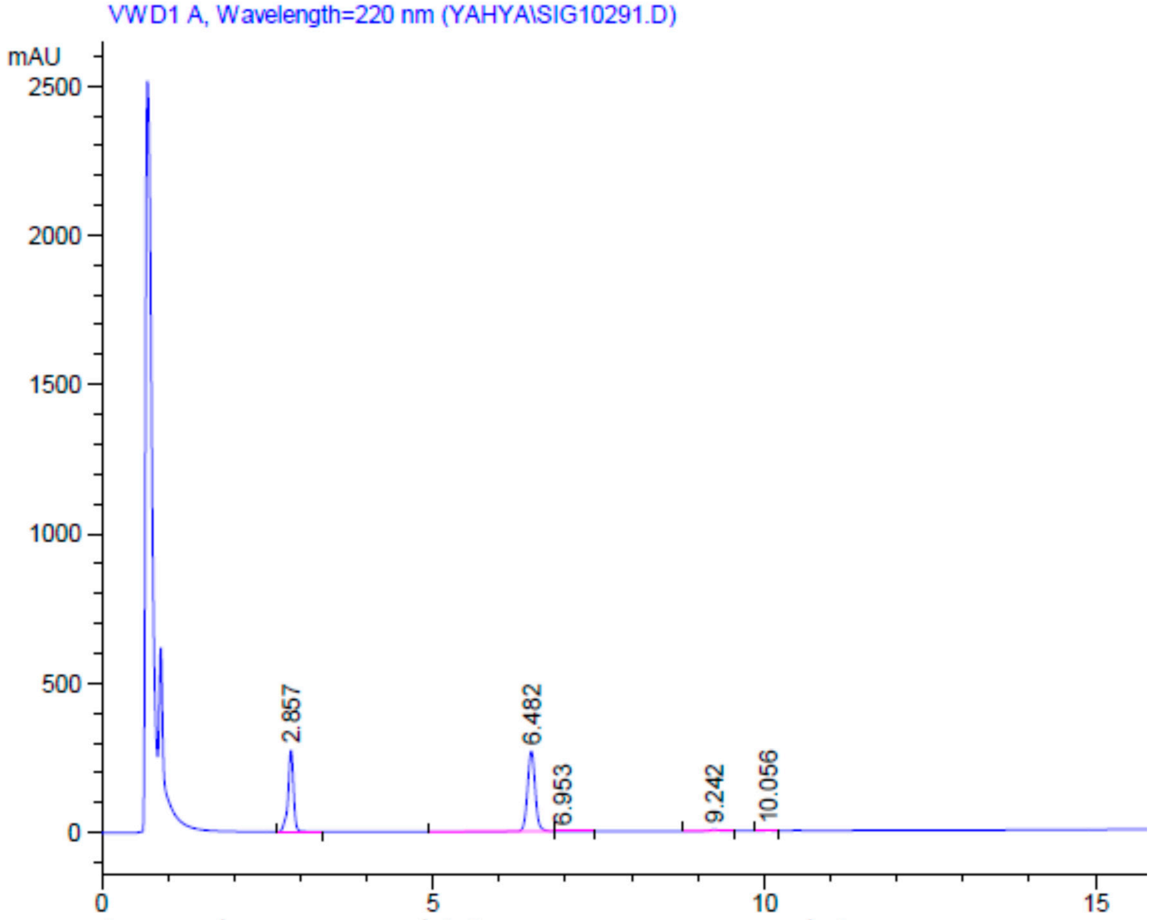

Peak #	RetTime [min]	Type	Width [min]	Area mAU *s	Height [mAU]	Area %
1	2.857	VV	0.0902	1638.37451	273.76172	41.7922
2	6.482	VV	0.1248	2204.88013	268.76138	56.2428
3	6.953	VB	0.2437	19.95272	1.14687	0.5090
4	9.242	BB	0.1372	41.32044	4.58364	1.0540
5	10.056	PV	0.1363	15.75997	1.76363	0.4020
Totals :				3920.28777	550.01724	

Figure S12. TOMBU.

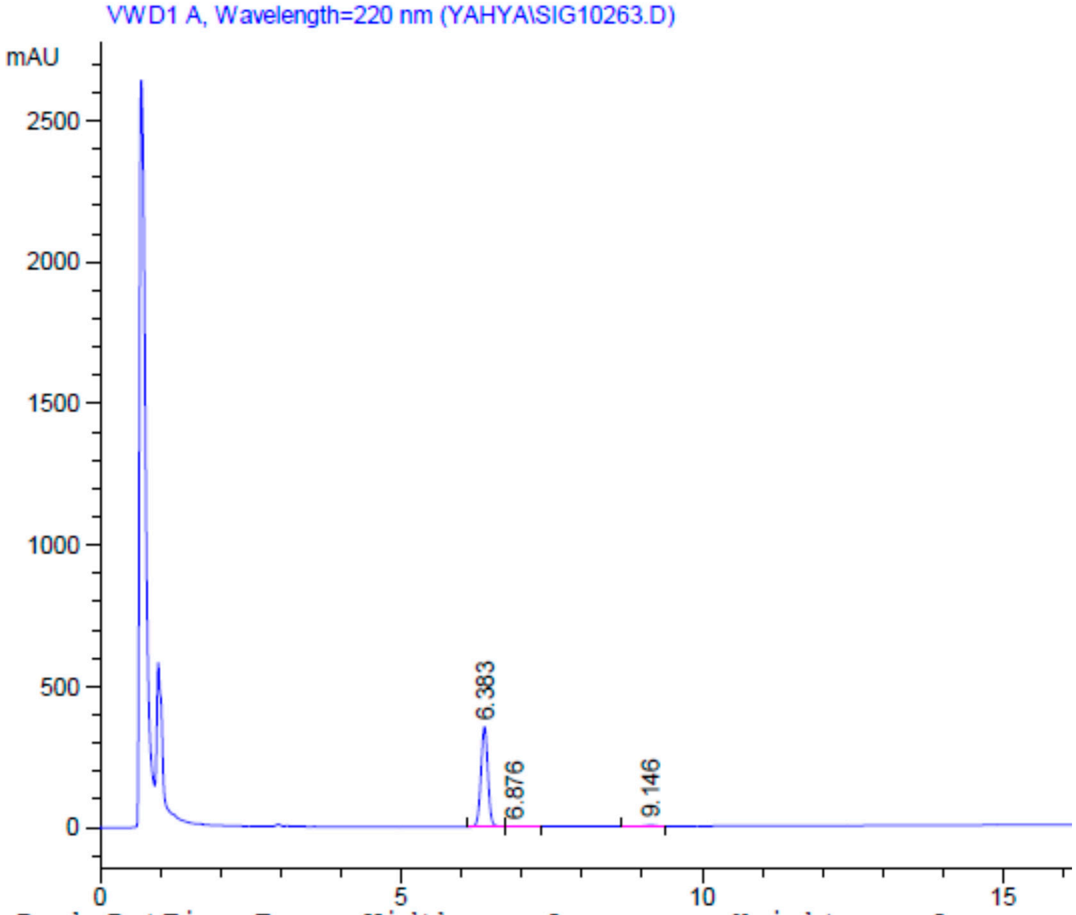

Peak #	RetTime [min]	Type	Width [min]	Area mAU *s	Height [mAU]	Area %
1	6.383	PV	0.1252	2822.78320	353.55396	97.8994
2	6.876	VB	0.2019	19.72105	1.48111	0.6840
3	9.146	BB	0.1535	40.84659	3.92370	1.4166

Totals : 2883.35085 358.95877

Figure S13. COMBU.

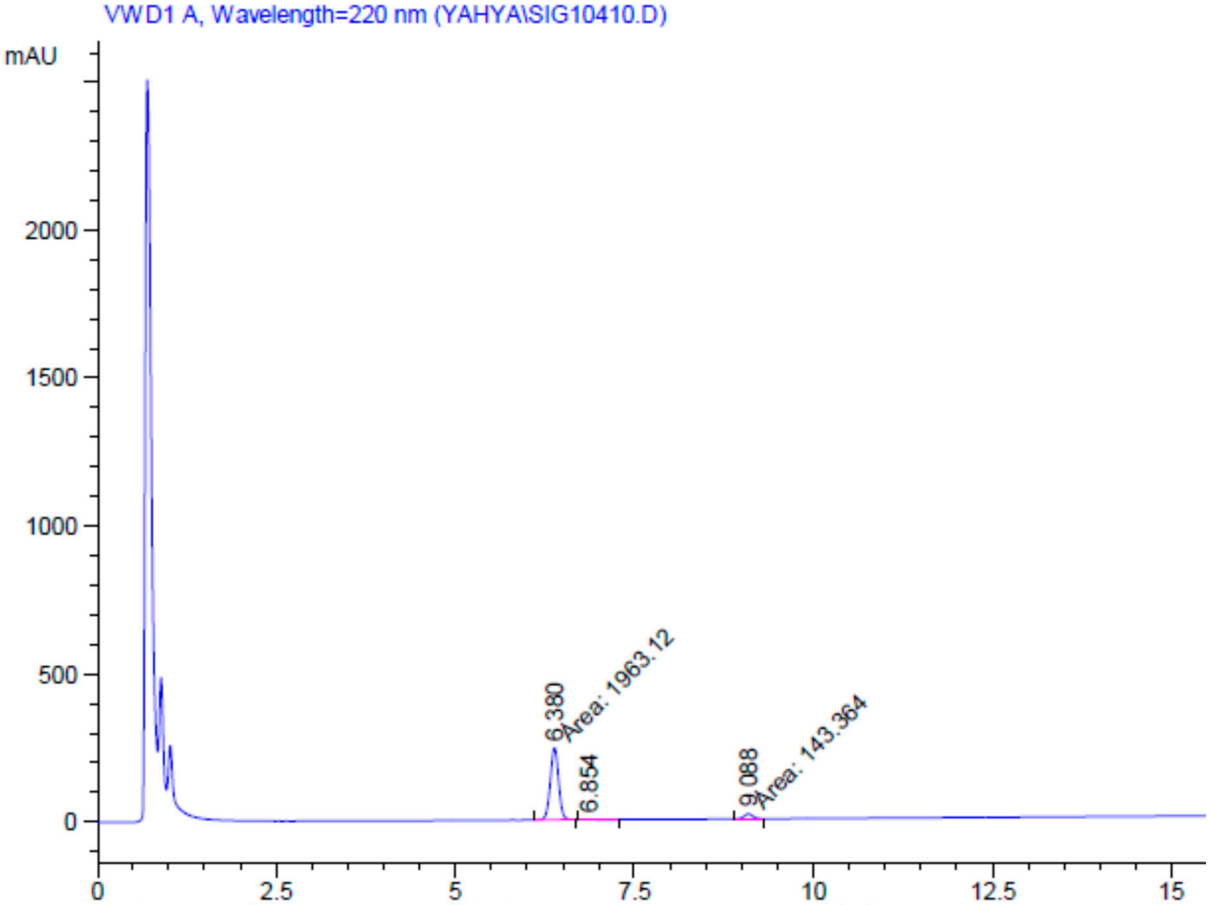

Peak #	RetTime [min]	Type	Width [min]	Area mAU *s	Height [mAU]	Area %
1	6.380	MM	0.1342	1963.12207	243.76393	92.3823
2	6.854	VB	0.1963	18.51307	1.38855	0.8712
3	9.088	MM	0.1448	143.36366	16.49790	6.7465
Totals :				2124.99881	261.65038	

2-Z-Phe-Val-Pro-NH_2 model

Figure S14. HBTU.

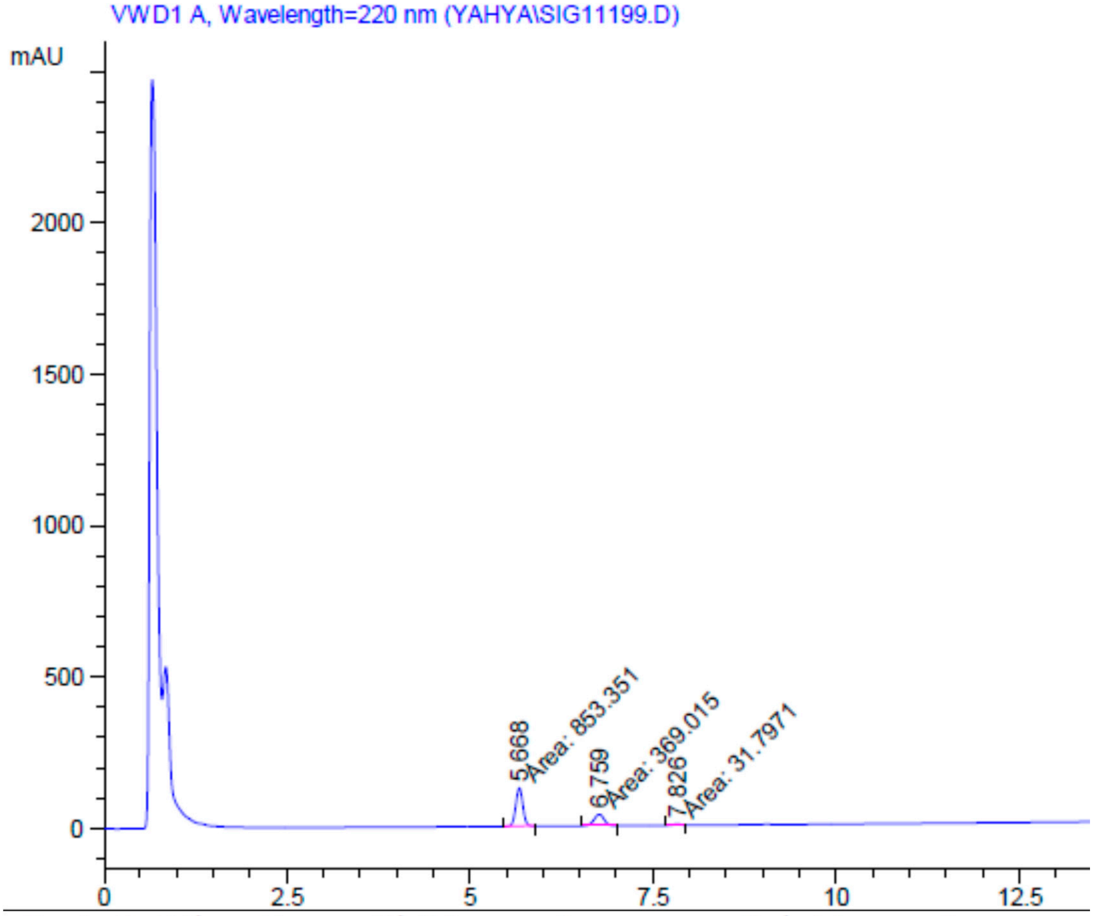

Peak #	RetTime [min]	Type	Width [min]	Area mAU *s	Height [mAU]	Area %
1	5.668	MM	0.1123	853.35095	126.61496	68.0415
2	6.759	MM	0.1619	369.01456	37.98602	29.4232
3	7.826	MM	0.1209	31.79707	4.38303	2.5353
Totals :				1254.16258	168.98401	

Figure S15. HATU.

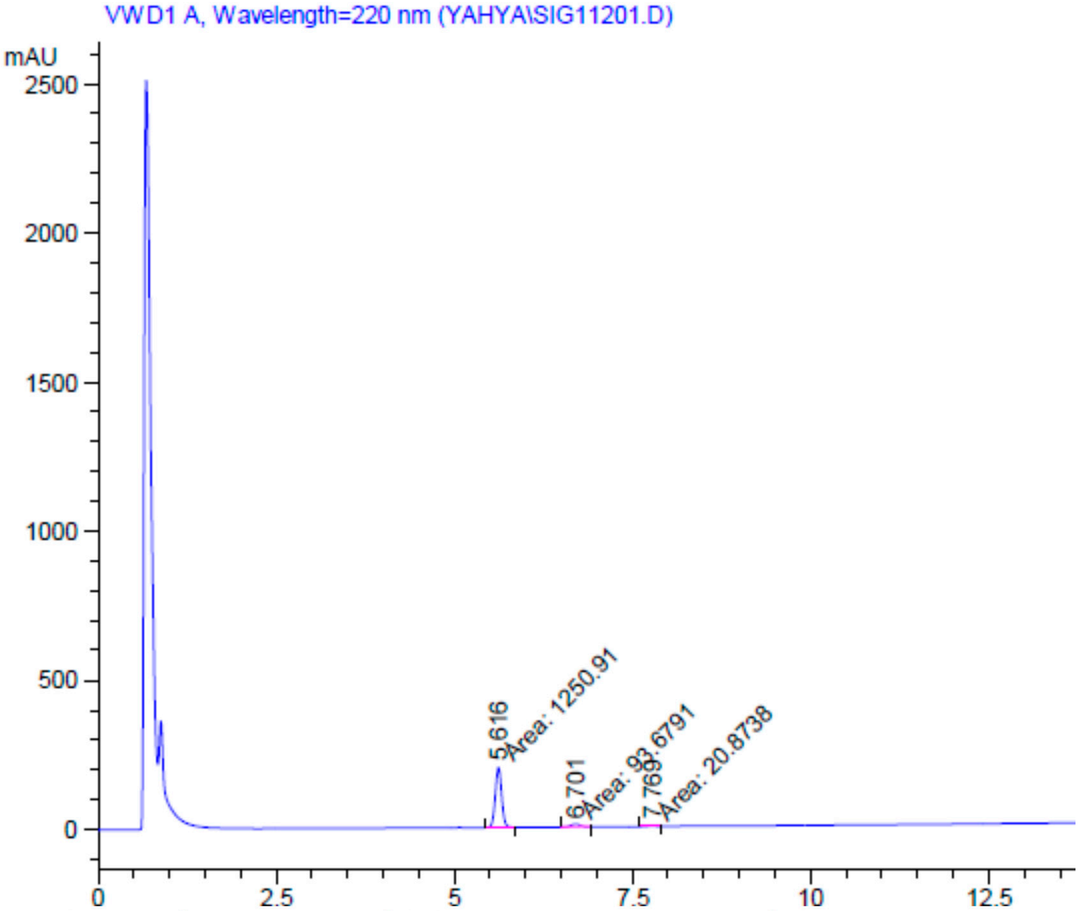

Peak #	RetTime [min]	Type	Width [min]	Area mAU *s	Height [mAU]	Area %
1	5.616	MM	0.1037	1250.91284	201.01862	91.6107
2	6.701	MM	0.1603	93.67911	9.73955	6.8606
3	7.769	MM	0.1381	20.87382	2.51859	1.5287

Totals : 1365.46577 213.27676

Figure S16. COMU.

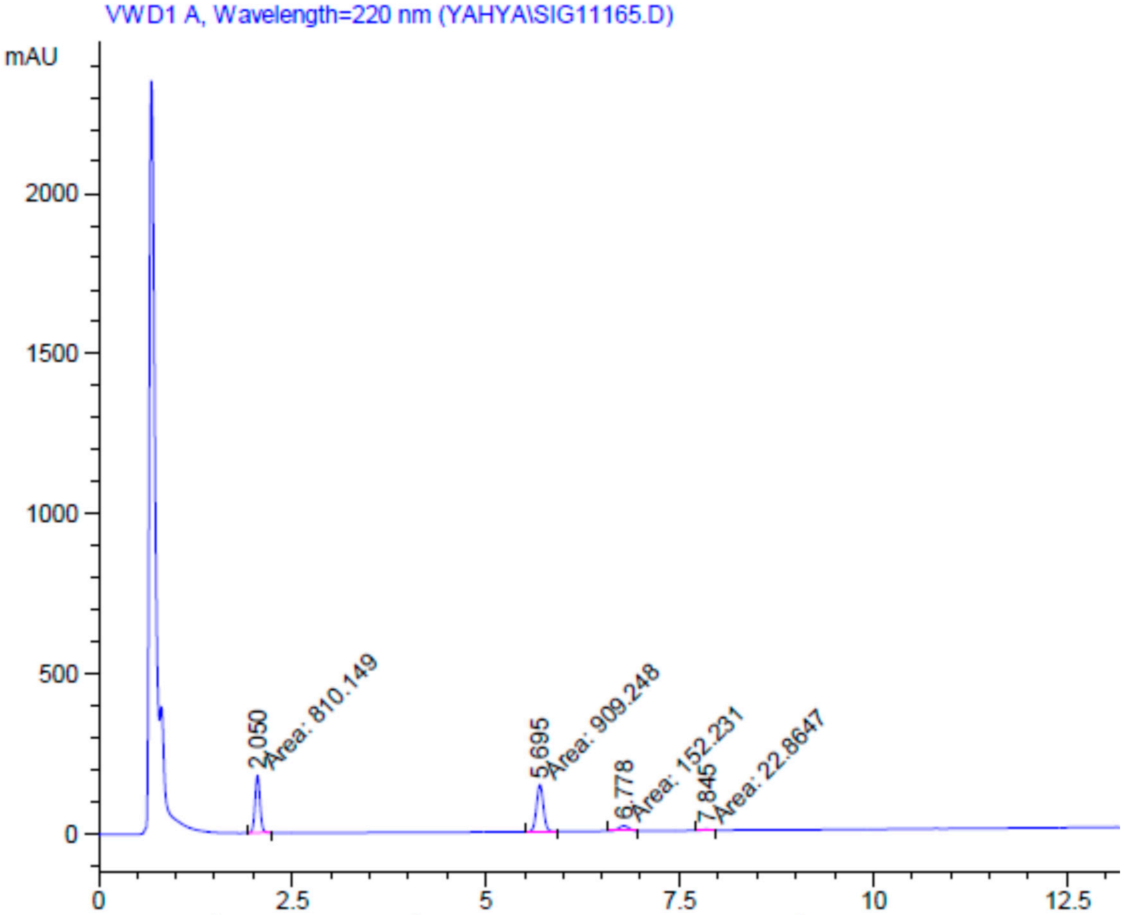

Peak #	RetTime [min]	Type	Width [min]	Area mAU *s	Height [mAU]	Area %
1	2.050	MM	0.0746	810.14923	180.88133	42.7634
2	5.695	MM	0.1030	909.24805	147.14796	47.9943
3	6.778	MM	0.1549	152.23134	16.37667	8.0355
4	7.845	MM	0.1253	22.86467	3.04160	1.2069

Totals : 1894.49328 347.44757

Figure S17. TOMBU.

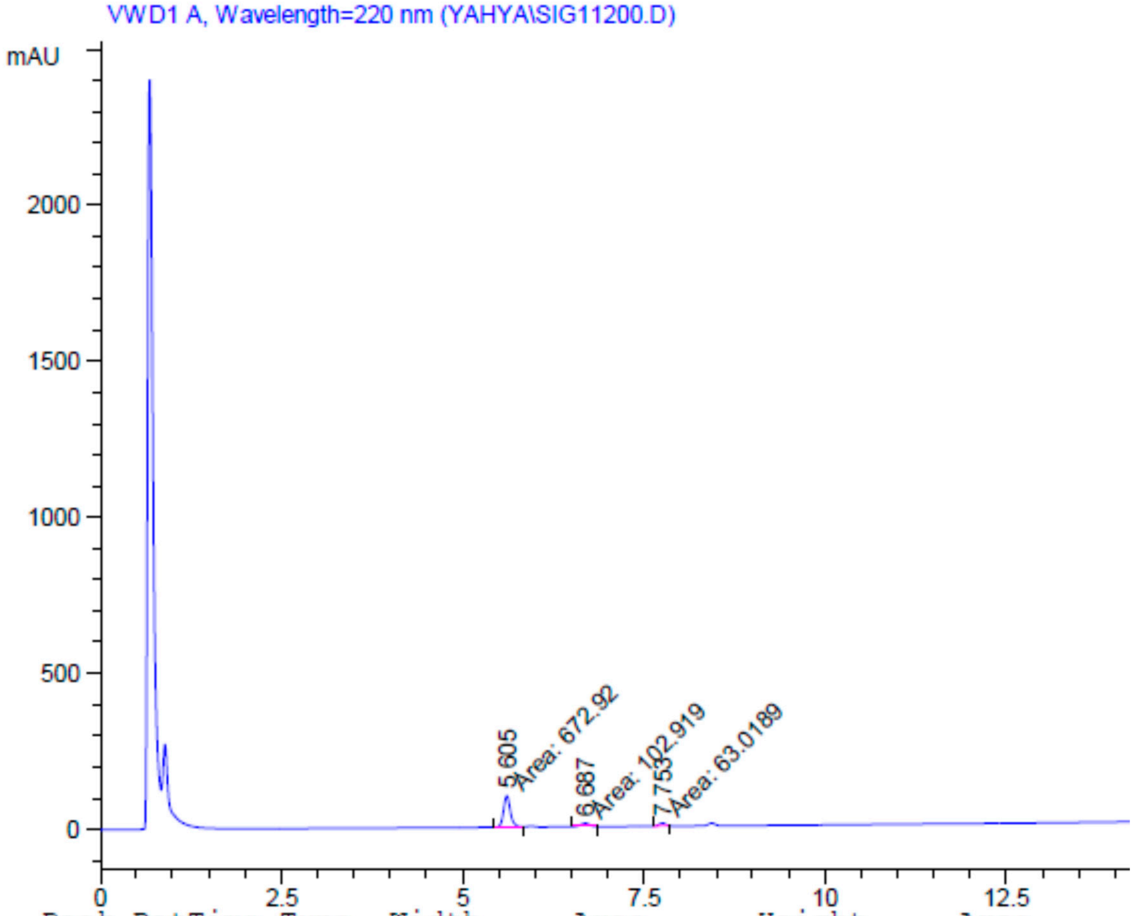

Peak #	RetTime [min]	Type	Width [min]	Area mAU *s	Height [mAU]	Area %
1	5.605	MM	0.1109	672.92010	101.16686	80.2186
2	6.687	MM	0.1539	102.91918	11.14570	12.2690
3	7.753	MM	0.1106	63.01891	9.49576	7.5125

Totals : 838.85819 121.80832

Figure S18. COMBU.

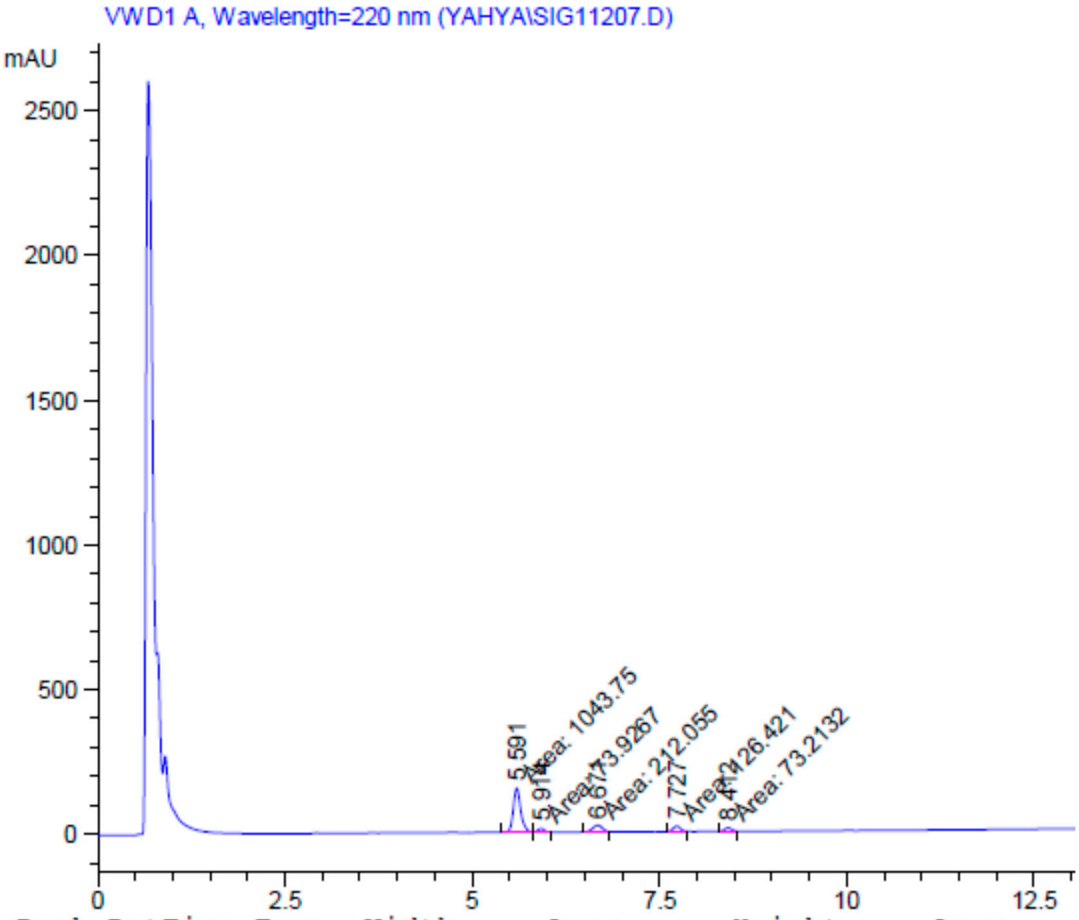

Peak #	RetTime [min]	Type	Width [min]	Area mAU *s	Height [mAU]	Area %
1	5.591	MF	0.1140	1043.75171	152.61850	68.2472
2	5.914	FM	0.1036	73.92673	11.89560	4.8338
3	6.671	MM	0.1520	212.05534	23.24917	13.8656
4	7.727	MM	0.1127	126.42139	18.70009	8.2662
5	8.412	MM	0.1033	73.21324	11.81439	4.7872
Totals :				1529.36841	218.27774	

3-H-Tyr-Aib-Aib-Phe-Leu-NH_2 model

Figure S19. HBTU.

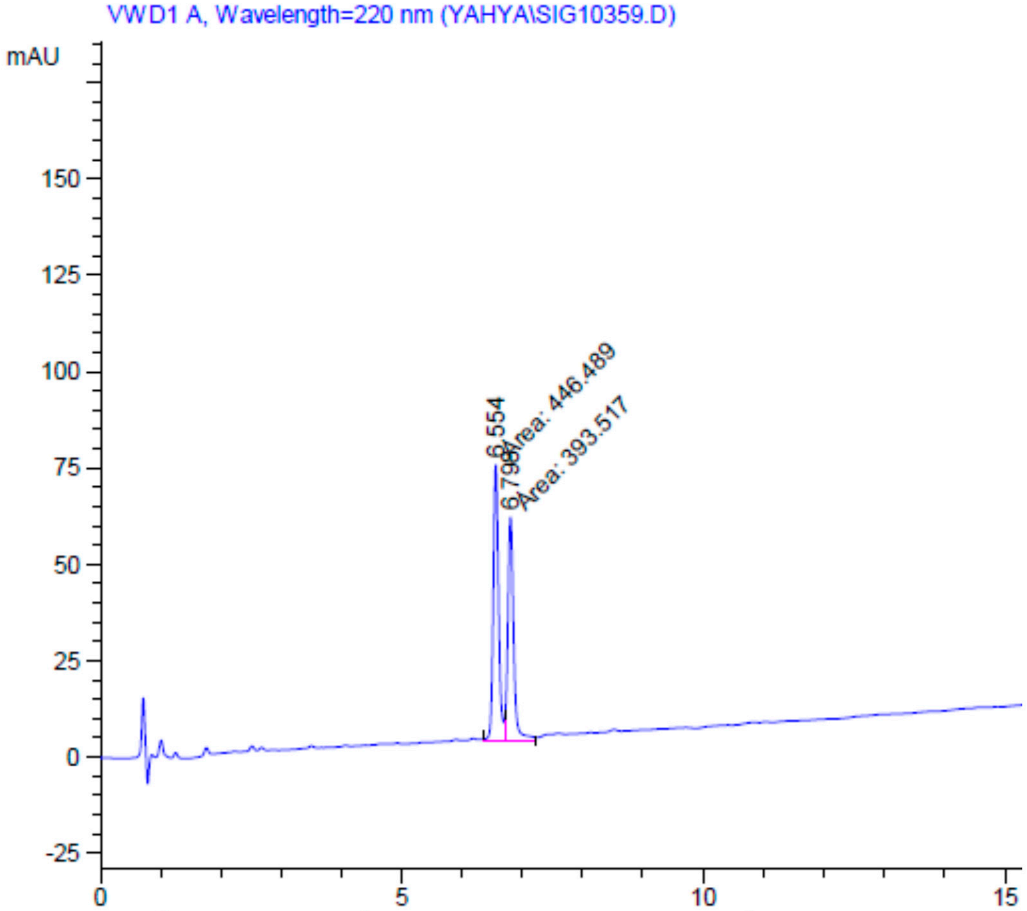

Peak #	RetTime [min]	Type	Width [min]	Area mAU *s	Height [mAU]	Area %
1	6.554	MF	0.1034	446.48859	71.94447	53.1531
2	6.798	FM	0.1125	393.51651	58.27957	46.8469
Totals :				840.00510	130.22404	

Figure S20. HATU.

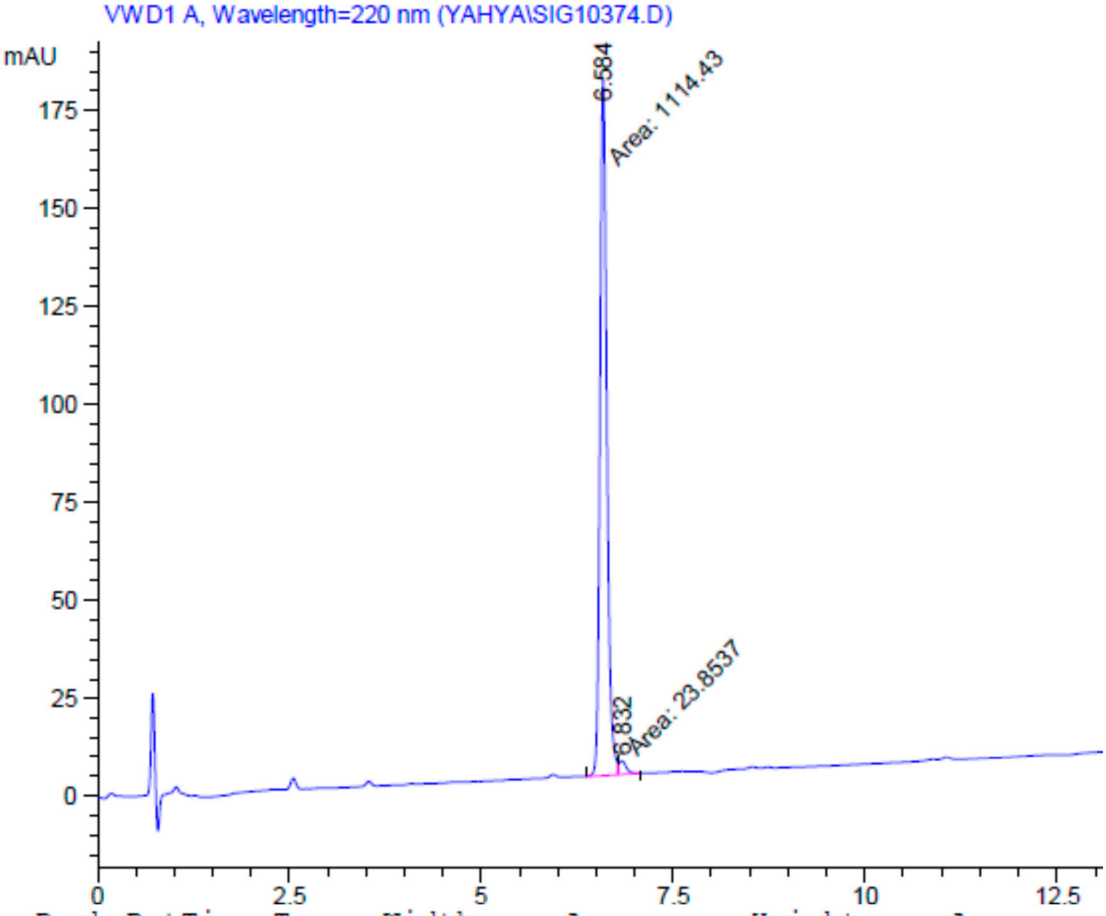

Peak #	RetTime [min]	Type	Width [min]	Area mAU *s	Height [mAU]	Area %
1	6.584	MF	0.1039	1114.42676	178.70680	97.9044
2	6.832	FM	0.1129	23.85373	3.51984	2.0956

Totals : 1138.28049 182.22665

Figure S21. COMU.

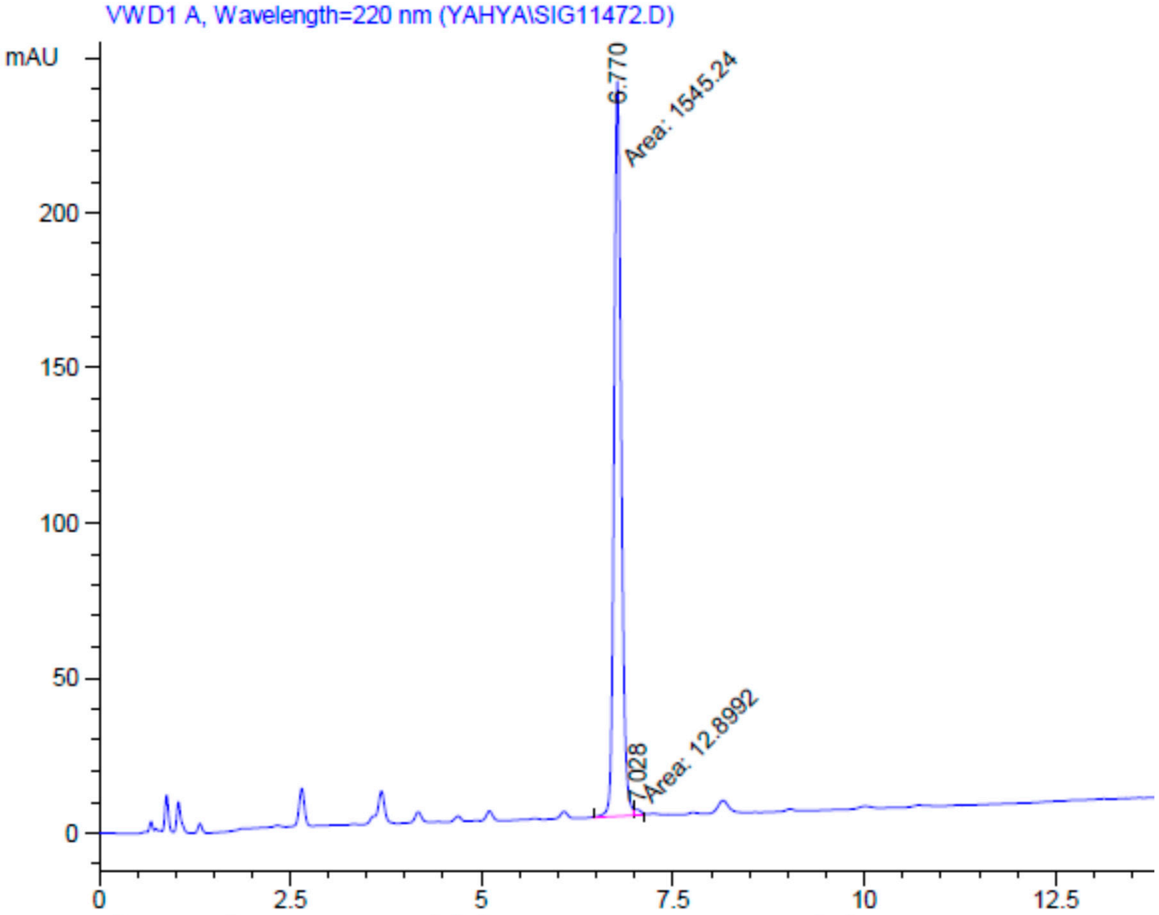

Peak #	RetTime [min]	Type	Width [min]	Area mAU *s	Height [mAU]	Area %
1	6.770	MF	0.1083	1545.23828	237.84361	99.1721
2	7.028	FM	0.1031	12.89924	2.08591	0.8279

Totals : 1558.13752 239.92952

Figure S22. TOMBU.

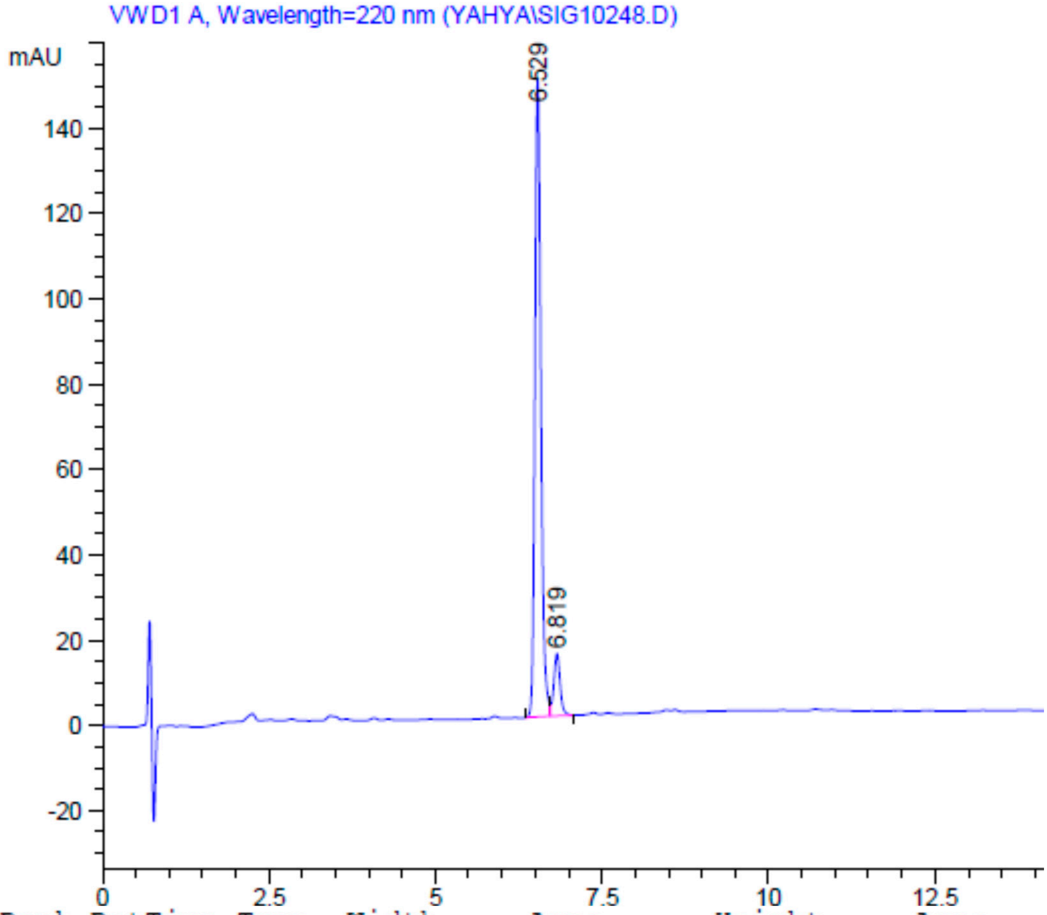

Peak #	RetTime [min]	Type	Width [min]	Area mAU *s	Height [mAU]	Area %
1	6.529	PV	0.0963	933.79376	149.22453	90.4376
2	6.819	VB	0.1017	98.73495	14.68465	9.5624

Totals : 1032.52871 163.90919

Figure S23. COMBU.

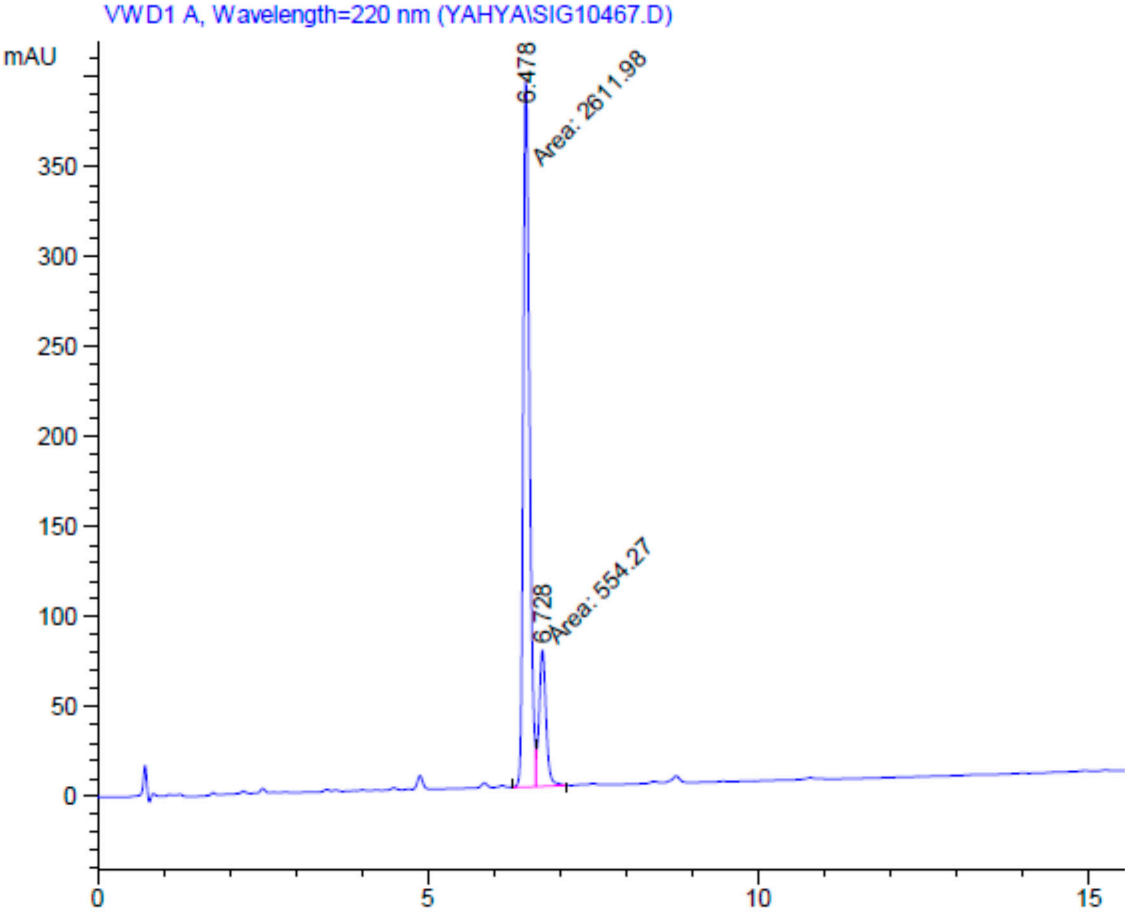

Peak #	RetTime [min]	Type	Width [min]	Area mAU *s	Height [mAU]	Area %
1	6.478	MF	0.1105	2611.98242	394.13702	82.4944
2	6.728	FM	0.1210	554.27002	76.36382	17.5056

Totals : 3166.25244 470.50084

Figure S24. COMBU (Fmoc-amino acids were preactivated with only 1 equiv. DIEA for 15–30 s, with addition of another 1 equiv. onto the resin after the first addition).

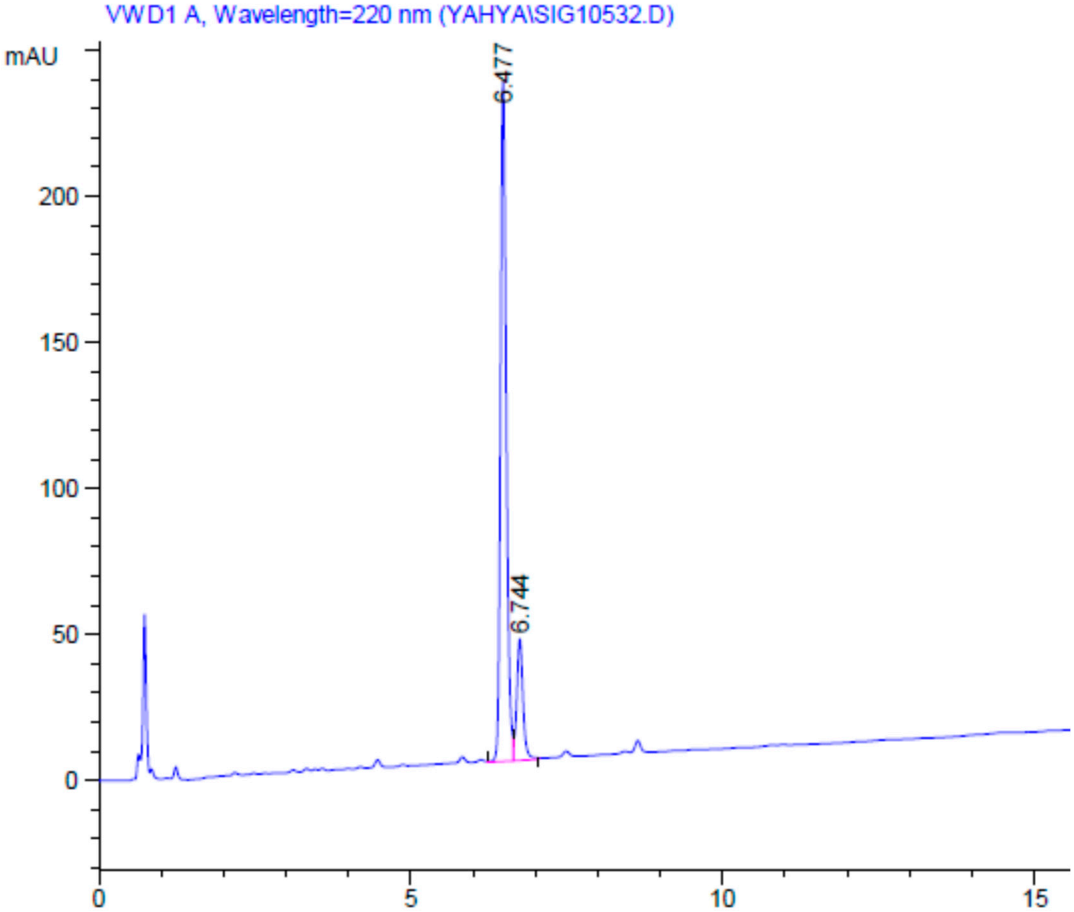

Peak #	RetTime [min]	Type	Width [min]	Area mAU *s	Height [mAU]	Area %
1	6.477	VV	0.1015	1506.33484	233.26291	83.5384
2	6.744	VB	0.1063	296.83081	41.71316	16.4616
Totals :				1803.16565	274.97607	
